# Stability of the surface diffusion flow and volume-preserving mean curvature flow in the flat torus

**DOI:** 10.1007/s00208-024-02863-3

**Published:** 2024-04-13

**Authors:** Daniele De Gennaro, Antonia Diana, Andrea Kubin, Anna Kubin

**Affiliations:** 1 CEREMADE Department, Université Paris-Dauphine, 1 Place du Maréchal de Lattre de Tassigny, 75775 Paris, France; 2 Dipartimento di Scienze Matematiche, Fisiche e Informatiche, Università degli Studi di Parma, Parco Area delle Scienze 53/A, 43124 Parma, Italy; 3 Scuola Superiore Meridionale, Largo San Marcellino 10, 80138 Naples, Italy; 4 Zentrum Mathematik-M7, Technische Universität München, Boltzmannstrasse 3, 85747 Garching, Germany; 5 Dipartimento di Scienze Matematiche “G. L. Lagrange”, Politecnico di Torino, Corso Duca degli Abruzzi, 24, 10129 Turin, Italy; 6 Institute of Analysis and Scientific Computing, Technische Universität Wien, Wiedner Haupstrasse 8-10, 1040 Vienna, Austria

**Keywords:** 53E10, 53E40, 49Q20, 35B40, 37E35

## Abstract

We prove that, in the flat torus and in any dimension, the volume-preserving mean curvature flow and the surface diffusion flow, starting $C^{1,1}$-close to a strictly stable critical set of the perimeter *E*,  exist for all times and converge to a translate of *E* exponentially fast as time goes to infinity.

## Introduction

In this paper we establish global in time existence and convergence towards equilibrium of two physically relevant volume-preserving geometric motions, namely the volume-preserving mean curvature flow and the surface diffusion flow.

On the one hand, the first one is the volume-preserving counterpart of the well-known mean curvature flow, and it is defined as a smooth evolution of sets $E_t$ governed by the law

0.1 $$\begin{aligned} V_t=-\textrm{H}_{E_t}+{{\bar{\textrm{H}}}}_{E_t}\quad \text {on }\partial E_t, \end{aligned}$$

where $V_t$ and $\textrm{H}_{E_t}$ are the outer normal velocity and the mean curvature of $\partial E_t,$ respectively, while $.$ The mean curvature flow is a famous evolution model, with far-reaching geometric and physical applications, which has a rich history dating back to its use in material science. One notable application is in physical systems involving multiple phases, such as the motion of grain boundaries in materials science, as first discussed by Mullins [38].

On the other hand, the surface diffusion flow is a smooth flow of sets $E_t$ evolving according to the law

0.2 $$\begin{aligned} V_t=\Delta _{E_t} \textrm{H}_{E_t}\quad \text {on }\partial E_t, \end{aligned}$$

where $\Delta _{E_t}$ denotes the Laplace–Beltrami operator on $\partial E_t.$ Similar to the mean curvature flow, the surface diffusion flow has important applications in material science, especially in physical systems with multiple phases. It has been proposed in the physical literature by Mullins [37] to model surface dynamics for phase interfaces when the evolution is governed by mass diffusion in the interface.

The volume preserving mean curvature flow can be seen as a simplified, second-order version of the surface diffusion flow as both flows share several common properties. Indeed, from the evolution laws (0.1) and (0.2) it follows that the volume of the evolving sets is preserved along the two flows, as can be easily seen from the following computation

$$\begin{aligned} \dfrac{\,\text {d}}{\,\text {d}t}|E_t|=\int _{\partial E_t}V_t\,\text {d}{\mathcal {H}}^{N-1}=0, \end{aligned}$$

the perimeter is decreasing, since the evolution (0.1) satisfies

$$\begin{aligned} \dfrac{\,\text {d}}{\,\text {d}t} P(E_t)=\int _{\partial E_t} V_t \textrm{H}_{E_t}\,\text {d}{\mathcal {H}}^{N-1}=\int _{\partial E_t} \left( \textrm{H}_{E_t} - {{\bar{\textrm{H}}}}_{E_t} \right) ^2\,\text {d}{\mathcal {H}}^{N-1}\le 0, \end{aligned}$$

and an integration by parts shows for (0.2) that

$$\begin{aligned} \dfrac{\,\text {d}}{\,\text {d}t} P(E_t)=\int _{\partial E_t} V_t \textrm{H}_{E_t}\,\text {d}{\mathcal {H}}^{N-1}= -\int _{\partial E_t} |\nabla \textrm{H}_{E_t}|^2\,\text {d}{\mathcal {H}}^{N-1}\le 0. \end{aligned}$$

Moreover, these two evolutions can be regarded (at least formally) as gradient flows of the perimeter according to suitable metrics. In particular, the mean curvature flow can be considered as (a volume preserving modification of) the $L^2$-gradient flow of the perimeter, while the surface diffusion can be interpreted as its $H^{-1}$-gradient flow.

In both cases, singularities may appear in a finite time even for initial smooth sets (see [35]), therefore in general only short-time existence results are available, see for instance [15, 25] for the mean curvature flow and [14] for the surface diffusion flow (see also [22] for the case of triple junction clusters). Because of the (formal) gradient flow structure of the two flows, it is reasonable to expect that if the initial set is sufficiently close to a stable point (or a local minimizer) *E* of the perimeter, then the flow exists for all times and asymptotically converges to *E*. We refer to this property as *dynamical stability.* We will properly define the notion of stability in Definition 1.1, however we can summarize it as follows: stable sets are sets whose boundary has constant mean curvature and positive definite second variation of the perimeter (i.e., they are “stable” for the perimeter functional). In this paper, we will focus on the flat torus ${\mathbb {T}}^N,$ which is particularly interesting due to the variety of possible limit points of the flows, namely periodic constant mean curvature hypersurfaces. In the Euclidean space only unions of balls have constant mean curvature, whereas the flat torus admits a much broader range of such surfaces. However, a full characterization of constant mean curvature hypersurfaces in ${\mathbb {T}}^N$ is not available in any dimension. In dimension $N=2,$ the only sets with constant mean curvature are discs and stripes (also called lamellae), while for $N\ge 3$ there exist many nontrivial examples, as stripes, cylinders and triply periodic surfaces known as gyroids.

The aforementioned approach of studying the dynamical stability of stable sets has been used in many instances in the literature. Concerning the surface diffusion, this method was employed in [2, 17, 18], where the authors considered the surface diffusion (also with an extra elastic term) and the Mullins–Sekerka flows in the 2, 3-dimensional flat torus (see also the survey [11]) and proved the dynamical stability of stable sets. It should be noted that the flows considered in these works include nonlocal terms, but their results also apply to the evolution driven solely by the perimeter energy. In the Euclidan setting, other results for the surface diffusion deal with the stability of balls [14, 29, 41], infinite cylinders [30], two-dimensional triple junctions [21], as well double bubbles [1, 20] (see also [29] for similar results in different settings).

Regarding the volume preserving mean curvature flow, recent progresses have been made in proving the dynamical stability of strictly stable sets in the 3-dimensional flat torus [39], while older results mainly concern convex sets, balls, or the 2-dimensional setting. The dynamical stability of balls has been proven in the Euclidean setting under various hypotheses on the dimension or on the initial set in [15, 19, 25, 31]. We refer also to [40], where global existence and convergence results for a large class of geometric evolution laws have been considered, relying on the concept of $L^p$-maximal regularity for quasilinear parabolic equations. Another interesting approach, up to now limited to the mean curvature flow, deals with the long-time behaviour of weak solutions of the flow, in particular the so-called flat flows [4, 32]. Flat flows are measure-theoretic weak solutions to the mean curvature flow arising as the limit of a discrete-in-time approximation, based on the minimizing movement scheme, as the time-step parameter tends to 0. The exponential convergence of flat flows to balls has been proved in [26] in ${\mathbb {R}}^2,$ while in [7] the authors deal with the anisotropic and crystalline mean curvature flow in ${\mathbb {R}}^N, N\ge 2,$ and for convex initial data, showing the asymptotic convergence to a Wulff shape. Concerning the time-discrete flows, in [10, 36], the asymptotic convergence to balls in ${\mathbb {R}}^N, N\ge 2,$ is shown in the classical and fractional settings, respectively. In [9], two of the authors prove the dynamical stability for the discrete flow of strictly stable sets in the flat torus of any dimension.

In the present paper we are able to prove in any dimensions the dynamical stability of strictly stable sets in the flat torus both for the surface diffusion flow and the volume preserving mean curvature flow. By assuming the initial set to be close in the $C^{1,1}$-topology to a strictly stable set, we obtain global existence and asymptotic convergence of both the flows to (a translated of) the underlying stable set. This is quite surprising for the surface diffusion flow, which is a fourth-order flow. Our main result is the following.

### Theorem 0.1

Let $E\subset {\mathbb {T}}^N$ be a strictly stable set and let $E_0 =E_{u_0} \subset {\mathbb {T}}^N$ be the normal deformation of *E* induced by $u_0 \in C^{1,1}(\partial E)$ (see Definition 1.2) with $|E_0|=|E|.$ There exists $\delta =\delta (E)>0$ such that if $\Vert u_0\Vert _{C^{1,1}(\partial E)}\le \delta ,$ then

(i) the volume-preserving mean curvature flow $E_t$ starting from $E_0$ (defined in (1.10)) exists smooth for all times $t\ge 0,$ and $E_t \rightarrow E +\tau$ as $t \rightarrow \infty ,$ for some $\tau \in {\mathbb {T}}^N,$ in $C^k$ for every $k\in {\mathbb {N}}$ exponentially fast; 
(ii) the surface diffusion flow $E_t$ starting from $E_0$ (defined in (1.17)) exists smooth for all times $t\ge 0,$ and $E_t \rightarrow E +\tau$ as $t \rightarrow \infty ,$ for some $\tau \in {\mathbb {T}}^N,$ in $C^k$ for every $k\in {\mathbb {N}}$ exponentially fast.

Where with exponentially fast we mean that the sets $E_t$ can be written as normal deformations of $E+\tau$ induced by functions $u(\cdot ,t) \in C^{\infty }(\partial E+\tau )$ such that

$$\begin{aligned} \Vert u(\cdot ,t)\Vert _{C^k(\partial E+\tau )}\le C_k e^{-C_k t} \quad \text {for} \ t>0. \end{aligned}$$

The main technical novelty of our argument is the use a quantitative Alexandrov-type inequality, which has been obtained by two of the authors in [9, Theorem 1.3] and is applied for the first time to a continuous-in-time setting, in this paper. This technique allows us to treat in a unified fashion both the geometric flows considered. However, it seems to be quite general, in the sense that it can be adapted to other gradient flows of the perimeter functional. For instance, we are confident that the Mullins–Sekerka flow or, more in general, fractional gradient flows of the perimeter could be treated analogously, provided one has sufficient control on the Schauder estimates for the linearized system governing the evolutions. This will be the subject of future investigations. Moreover, since this stability inequality can be seen as a Łojasiewicz–Simon inequality with sharp exponents, one is able to derive the optimal decay of the dissipation along the flow, immediately yielding the exponential convergence in any norm of the flow to the subjacent strictly stable set. In particular, our line of proof works in any dimension without the need of deriving energy estimates for the high derivatives of the curvature, which was one the main bottleneck of the previous methods developed in [2, 17, 18]. Lastly, the Schauder-type estimates we provide following the lines of [24] seems to be new in this setting.

We now outline the strategy of the proof, which is based on the gradient flow structure of the evolution. Firstly, applying the Alexandrov-type inequality [9, Theorem 1.3], combined with the quantitative isoperimetric inequality of [3], we are able to bound the velocity in terms of the displacement. By iterating this procedure for the whole time of existence and using higher order estimates, we can extend the flow for all times. In order to do so, we need to show that the short-time existence and regularity results depend only on the bounds of the initial datum. This is not a priori clear from previous existence results [14, 15]. More precisely, we rely on Schauder estimates on the linearized problem solved by the flows, which is a quasilinear perturbation of the heat equation for the mean curvature flow and a quasilinear perturbation of the biharmonic heat equation for the surface diffusion flow. While Schauder-type estimates for general quasilinear parabolic PDEs of the second order are well known (see for instance [16]), we couldn’t find a precise reference for the fourth-order equation. Although an approach by scaling (in the spirit of [28]) could be feasible by working in local coordinates, we preferred to rely on the estimates provided in [24], where time-weighted Hölder norms are employed. After establishing the global existence of both flows, we obtain the exponential convergence up to translations via a Gronwall-type inequality. This is where it comes into play the optimality of the exponent in the Alexandrov theorem [9, Theorem 1.3], which yields the exponential rate of convergence. Finally, we prove the convergence of these translations by exploiting the decay of geometric quantities along the flow, as in [2].

We conclude by highlighting that a similar stability result for the surface diffusion flow has been obtained by the second author and collaborators in [13] using different techniques (that are shown in details in dimension $N=4$ and listed for any general *N*), assuming different hypotheses on the initial datum, depending on the dimension *N*. In particular, they consider initial sets $E_0$ close to the strictly stable set in $C^1$ and such that the energy

$$\begin{aligned} \int _{\partial E_0} |\nabla ^{N-2}{\textrm{H}}|^2 \,\text {d}{\mathcal {H}}^{N-1} + \int _{\partial E_0} |\nabla {\textrm{H}}|^2 \,\text {d}{\mathcal {H}}^{N-1} \end{aligned}$$

is sufficiently small.

## Preliminary results

In this section we collect some preliminary results and we fix the notations.

We denote by ${\mathbb {T}}^N$ the *N*-dimensional flat torus, which is the quotient of ${\mathbb {R}}^N$ by ${\mathbb {Z}}^N.$ The function spaces $C^k({\mathbb {T}}^N)$ and $W^{k,p}({\mathbb {T}}^N),$ for $k \in {\mathbb {N}}$ and $p \in [1,\infty ],$ are defined as the restriction of $C^k({\mathbb {R}}^N)$ and $W_{loc}^{k,p}({\mathbb {R}}^N),$ respectively, to the functions that are one-periodic. With $B_r(x)$ we denote the ball in ${\mathbb {R}}^N$ of center *x* and radius *r*,  while $B_r$ will be a short-hand notation for $B_r(0).$ Given $x \in {\mathbb {R}}^N,$ we will write $x=(x',x_N)$ where $x'\in {\mathbb {R}}^{N-1}$ and $x_N\in {\mathbb {R}}.$ Similarly, we denote by $B'_r(x')\subset {\mathbb {R}}^{N-1}$ the ball in ${\mathbb {R}}^{N-1}$ with radius $r>0$ and center $x' \in {\mathbb {R}}^{N-1}.$

Moreover, we denote by *c*, *C* some constants, which could be changing from line to line and always depend on the dimension *N*,  and by $\frac{\partial }{\partial t}$ (or equivalently $\partial _t$) the partial derivative with respect to the variable *t*. Let $F \subset {\mathbb {T}}^N$ we denote with $\text {dist}_F(\cdot )$ the distance from the set *F* and with $C^{1,1}(\partial F)$ the set of functions continuously differentiable with derivative Lipschitz continuous on $\partial F.$

Given a smooth closed $(N-1)$-manifold $\Sigma \subset {\mathbb {T}}^N$ we denote by $\nu _{\Sigma }:\Sigma \rightarrow {\mathbb {S}}^N$ the outer normal to $\Sigma ,$ by $B_{\Sigma }$ the second fundamental form of $\Sigma ,$ and by $\textrm{H}_{\Sigma }$ its mean curvature, that is the trace of $B_{\Sigma }.$ For every vector field $X:\Sigma \rightarrow {\mathbb {R}}^N$ we let $X_\tau$ to be the tangential part of *X*,  that is $X_\tau (x)=X(x)-X(x) \cdot \nu _{\Sigma }(x) \nu _{\Sigma }(x),$ and for every function $f \in L^1(\Sigma )$ we denote with

$$\begin{aligned} {\bar{f}}= \frac{1}{{\mathcal {H}}^{N-1}(\Sigma )}\int _{\Sigma } f d {\mathcal {H}}^{N-1} \end{aligned}$$

the mean of *f* over $\Sigma .$

Let $E \subset {\mathbb {T}}^N$ be a open set with smooth boundary and let $X:{\mathbb {T}}^N\rightarrow {\mathbb {R}}^N$ be a vector field of class $C^2.$ We consider the associated flow $\Phi :{\mathbb {T}}^N\times (-1,1)\rightarrow {\mathbb {T}}^N$ defined by

1.1 $$\begin{aligned} \frac{\partial \Phi }{\partial t}=X(\Phi ) \quad \Phi (\cdot ,0)=I, \end{aligned}$$

where $I:{\mathbb {T}}^N \rightarrow {\mathbb {T}}^N$ denotes the identity, and we say that $E_t =\Phi (E,t)$ is the variation of *E* associated to $\Phi$ (or to *X*). If in addition it holds $\vert E_t \vert = \vert E \vert$ for every $t \in (-1,1),$ we say that $E_t$ is a *volume-preserving variation* of *E*.

We now recall some results on sets of finite perimeter, referring to [33] for the basic definitions and proofs. We say that a measurable set $E \subset {\mathbb {T}}^N$ is a set of finite perimeter if

1.2 $$\begin{aligned} P(E):=\sup \left\{ \int _{E} \text {div}(X)\,\text {d}x : X \in C^1({\mathbb {T}}^N,{\mathbb {R}}^N),\, \vert X \vert \le 1 \right\} <\infty . \end{aligned}$$

Moreover, by De Giorgi’s structure theorem, we have $P(E)= {\mathcal {H}}^{N-1}(\partial ^* E)$ where $\partial ^* E$ is a suitable $(N-1)$-rectifiable subset of $\partial E.$ The first and second variation of the perimeter at *E* with respect to the flow $\Phi$ are defined as follows

$$\begin{aligned} \delta P(E)[X]:=\dfrac{\,\text {d}}{\,\text {d}t}\Big |_{t=0}P(E_t), \quad \delta ^2 P(E)[X]:=\dfrac{\,\text {d}^2}{\,\text {d}t^2}\Big |_{t=0}P(E_t) . \end{aligned}$$

It is well known that, for any set of finite perimeter *E*,  we have

$$\begin{aligned} \delta P(E)[X]=\int _{\partial ^* E } \text {div}_\tau (X)d{\mathcal {H}}^{N-1} , \end{aligned}$$

where $\text {div}_{\tau }(X)$ is the tangential divergence of *X* on *E* and it is given by

1.3 $$\begin{aligned} \text {div}_{\tau }(X)(x)= \text {div}(X)(x)-\nu _{E}(x) \cdot \nabla X(x) \quad \text {for all } x \in \partial ^* E. \end{aligned}$$

Moreover, if *E* is a open set with $C^2$-boundary we have

$$\begin{aligned} \delta P(E)[X]=\int _{\partial E } \text {div}_\tau (X)d{\mathcal {H}}^{N-1} =\int _{\partial E} \textrm{H}_E \nu _E\cdot X\,\text {d}{\mathcal {H}}^{N-1}. \end{aligned}$$

Finally, the second variation formula for perimeter on open sets of class $C^2$ (see for instance [3, Section 3]) is given by

$$\begin{aligned}&\delta ^2 P(E)[X]=\int _{\partial E} \left( | {\nabla _\tau } (X\cdot \nu _E)|^2-|B_E|^2 (X\cdot \nu _E)^2 \right) \,\text {d}{\mathcal {H}}^{N-1}\\&\quad -\int _{\partial E} \textrm{H}_E \text {div}_\tau (X_\tau (X\cdot \nu _E)) \,\text {d}{\mathcal {H}}^{N-1}+\int _{\partial E} \textrm{H}_E(\text {div}X)(X\cdot \nu _E) \,\text {d}{\mathcal {H}}^{N-1}, \end{aligned}$$

where $\nabla _\tau f(x)= (\nabla f)_{\tau }(x)$ denotes the tangential derivative of *E*. Since the expression above only depends on the normal component of the velocity field *X*,  we also denote by $\delta P(E)[\varphi ]$ and $\delta ^2 P(E)[\varphi ],$ respectively, the first and the second variation of the perimeter at *E*,  where $\varphi = X \cdot \nu _E.$

Let *E* be a critical point of the perimeter. It should be noted that the translation invariance of the perimeter implies that the second variation becomes degenerate along flows of the form $\Phi (x,t)=x+t\eta ,$ where $\eta \in {\mathbb {R}}^N.$ Because of that, we denote by

$$\begin{aligned} {{\tilde{H}}}^1(\partial E):=\left\{ \varphi \in H^1(\partial E) : \int _{\partial E} \varphi \,\text {d}{\mathcal {H}}^{N-1}=0 \right\} , \end{aligned}$$

and by $T(\partial E)$ the subspace generated by the functions $\nu _i : \partial E \rightarrow {\mathbb {R}}$ for $i=1,\ldots , N,$ defined as $\nu _i:=e_i \cdot \nu _E$ where $e_1,\ldots , e_N$ is the standard orthonormal basis of ${\mathbb {R}}^N.$ We then set $T^\perp (\partial E)$ to be the orthogonal subspace of $T(\partial E)$ in the $L^2-$sense, that is

$$\begin{aligned} T^\perp (\partial E)= \left\{ \varphi \in {{\tilde{H}}}^1(\partial E) : \int _{\partial E} \varphi \nu _i\,\text {d}{\mathcal {H}}^{N-1}=0,\quad i=1,\ldots ,N \right\} . \end{aligned}$$

After defining all the spaces, we can finally give the notion of stability.

### Definition 1.1

We say that a set $E \subset {\mathbb {T}}^N$ of class $C^2$ is a *strictly stable set* if it is a critical set, that is $\delta P(E)[\varphi ]=0$ for all $\varphi \in {{\tilde{H}}}^1 (\partial E),$ and the second variation of the perimeter is positive definite, in the sense that

$$\begin{aligned} \delta ^2P(E)[\varphi ]>0,\quad \forall \varphi \in T^{\perp } (\partial E) \setminus \{0\}. \end{aligned}$$

We now recall some technical results that will be useful in the following. We start by recalling the definition of normal deformation of a set and a result which ensures that any $W^{2,p}$-small normal deformation of a smooth set can be translated in a way so the projection on the subspace $T^\perp (E)$ becomes small.

### Definition 1.2

Let $E \subset {\mathbb {T}}^{N}$ be an open set of class $C^{1}.$ For every $f\in L^{\infty }(\partial E)$ such that $\Vert f \Vert _{L^{\infty }(\partial E)}$ is sufficiently small, we define the *normal deformation of* *E* *induced by* *f* the set $E_f$ having as boundary

$$\begin{aligned} \partial E_{f}: =\left\{ x+ f(x)\nu _{E}(x) \, :\, x \in \partial E \right\} . \end{aligned}$$

### Lemma 1.3

[3, Lemma 3.8] Let $E\subset {\mathbb {T}}^N$ be of class $C^3$ and let $p>N-1.$ For every $\delta ^*>0$ there exist $C>0$ and $\eta >0$ such that if *F* is a normal deformation of *E* induced by some $\psi \in C^2({\partial E})$ with $\Vert \psi \Vert _{W^{2,p}(\partial E)}\le \eta ,$ that is $F=E_\psi ,$ then there exist $\sigma \in {\mathbb {T}}^N$ and $\varphi \in W^{2,p}(\partial E)$ with the properties that

$$\begin{aligned} |\sigma |\le C\Vert \psi \Vert _{W^{2,p}(\partial E)},\quad \Vert \varphi \Vert _{W^{2,p}(\partial E)}\le C\Vert \psi \Vert _{W^{2,p}(\partial E)} \end{aligned}$$

and

$$\begin{aligned} F+\sigma =E_\varphi , \quad \left|\int _{\partial E}\varphi \nu _E\,\text {d}{\mathcal {H}}^{N-1}\right|\le \delta ^* \Vert \varphi \Vert _{L^2(\partial E)}. \end{aligned}$$

We now recall the definition of inner and outer ball condition.

### Definition 1.4

We say that a open set $E \subset {\mathbb {T}}^N$ satisfies a *uniform inner (respectively outer) ball condition* with radius *r* if there exists $r>0$ such that for every $x \in \partial E$ there exists a ball $B_r(y) \subset E$ (resp. $B_r(y) \subset E^c$) with $x \in \partial B_r(y).$

Note that all sets $$E\subset {\mathbb {T}}^N$$ of class $$ C^{1,1}$$ satisfy a uniform inner and outer ball condition (see e.g. [8]). Arguing as in the proof of [3, Lemma 3.8], we can prove the following result.

### Lemma 1.5

Let $E \subset {\mathbb {T}}^N$ be of class $C^{\infty }$ and let $m>0.$ There exists $\eta =\eta (m,E)>0$ such that,  for every $k\in {\mathbb {N}},$ $u\in C^k(\partial E)$ with $\Vert u\Vert _{C^k(\partial E)}\le m,$ $\Vert u\Vert _{C^0(\partial E)}\le \eta$ and for every $\sigma \in {\mathbb {T}}^N$ with $|\sigma |\le \eta ,$ then $E_u+\sigma$ can be written as a normal deformation of *E* induced by a function $v:\partial E\rightarrow \partial E$ such that

$$\begin{aligned} \Vert v\Vert _{C^0(\partial E)}\le 2 \eta , \quad \Vert v\Vert _{C^k(\partial E)}\le C (\Vert u\Vert _{C^k(\partial E)}+|\sigma |), \end{aligned}$$

where $$C=C(E)>0.$$

### Proof

Being the set *E* smooth, it satisfy the uniform inner and outer ball condition, hence there exists a positive radius $r>0$ such that the signed distance $\text {sd}_E$ from the set *E*,  defined by

$$\begin{aligned} \text {sd}_E(x)= {\left\{ \begin{array}{ll} \text {dist}_{\partial E}(x) &{}\hbox {if}\ x \in E^c\\ -\text {dist}_{\partial E}(x) &{}\text {if }x \in E, \end{array}\right. } \end{aligned}$$

is a function of class $C^{\infty }$ (from the regularity of $\partial E$) in the *r*-tubular neighborhood $(\partial E)_{r},$ that is $(\partial E)_r :=\left\{ x :\, \text {dist}_{\partial E}(x)<r\right\}$ (for further properties of the distance function see [23, section 14.6]). Since, for some $k\ge 2,$ *u* has $C^k$-norm bounded by *m*,  we also have $\Vert u\Vert _{C^{1,1}(\partial E)}\le m.$ Then, there exists a radius $\rho =\rho (m,E)$ such that $\partial E_u$ satisfies a uniform inner and outer ball condition of radius $\rho .$ We can assume without loss of generality that $\rho <r.$

We now let $\eta \le \rho /2$ to be chosen later, take any $|\sigma |< \eta$ and set $F=E_u+\sigma .$ Clearly, *F* still satisfies a uniform inner and outer ball condition of radius $\rho .$ Then, for every $y\in \partial F$ there exists $x\in \partial E_u$ such that $y=x+\sigma ,$ hence we have

$$\begin{aligned} \text {dist}_{\partial E}(y)\le |\sigma |+\text {dist}_{\partial E}(x)< \eta +\Vert u\Vert _{C^0(\partial E)}\le 2\eta , \end{aligned}$$

and in particular $\partial F\subset (\partial E)_{2 \eta } \subset (\partial E)_r.$ We now define the map $T_u:\partial E\rightarrow \partial E$ as

1.4 $$\begin{aligned} T_u(x):=\pi _{ E}(x+u(x)\nu _E(x)+\sigma )= y-\text {sd}_E(y) \nabla \text {sd}_E(y), \end{aligned}$$

where $\pi _{E}$ is the projection map on $\partial E$ and $y=x+u(x)\nu _E(x)+\sigma \in \partial F.$ By choosing $\eta$ smaller, by interpolation, it holds $\Vert u\Vert _{C^1(\partial E)} + | \sigma | < \frac{1}{2} ,$ which implies that the function $x \mapsto x+ u(x)\nu _{E}(x)+\sigma$ is a diffeomorphism (since it is a small perturbation of the identity). Moreover, since *E* is of class $C^{\infty }$ (and possibly for $\eta$ smaller), $\pi _{ E} \big |_{\partial F}:\partial F \rightarrow \partial E$ is a diffeomorphism of class $C^k,$ $C^k$-close to the identity. Therefore, $T_u\in C^k(\partial E)$ and, by (1.4), we get

1.5 $$\begin{aligned} \Vert T_u-I\Vert _{C^k(\partial E)}\le C(\Vert u\Vert _{C^k(\partial E)}+|\sigma |). \end{aligned}$$

Moreover, using again (1.4) and the invertibility of the map $x \mapsto x+u(x)\nu _E(x)+\sigma ,$ we obtain

1.6 $$\begin{aligned} \Vert T^{-1}_u-I\Vert _{C^k(\partial E)}\le C(\Vert u\Vert _{C^k(\partial E)}+|\sigma |). \end{aligned}$$

Using the fact that $T_u$ is a diffeomorphism and (1.4), we can find a function $v:\partial E\rightarrow {\mathbb {R}}$ such that *F* is the normal deformation of *E* induced by *v*,  more precisely for every $x\in \partial E$ it holds

$$\begin{aligned} x+u(x)\nu _E(x)+\sigma =T_u(x)+v(T_u(x))\nu _E(T_u(x)). \end{aligned}$$

Finally, using the above expression and the bounds in (1.5) and (1.6), we conclude that

$$\begin{aligned} \Vert v\Vert _{C^k(\partial E)}\le & {} \Vert T_u^{-1}\Vert _{C^k(\partial E)}(\Vert u\Vert _{C^k(\partial E)}+|\sigma |+\Vert T_u-I\Vert _{C^k(\partial E)})\\\le & {} C(\Vert u\Vert _{C^k(\partial E)}+|\sigma |), \end{aligned}$$

for some constant $C=C(E)>0.$ $\quad \square$

Let $E, F\subset {\mathbb {T}}^N$ be measurable sets. We define a $L^1$-distance between *E*, *F* modulo translations (also known as the Fraenkel asymmetry of the set *E* related to *F*) as

$$\begin{aligned} \alpha (E,F) :=\min _{x \in {\mathbb {T}}^N} |E \triangle (F+x)|. \end{aligned}$$

The following quantitative isoperimetric inequality has been proved in [3]. As a consequence of this result, strictly stable sets are of class $C^{\infty }$ (see [33]).

### Theorem 1.6

[3, Corollary 1.2] Let $E \subset {\mathbb {T}}^N$ be a strictly stable set. Then,  there exist $\eta = \eta (E),$ $C=C(E)>0$ such that

$$\begin{aligned} C\alpha ^2(E,F)\le P(F)-P(E) \end{aligned}$$

for all $F\subset {\mathbb {T}}^N$ with $|F|=|E|$ and $\alpha (E,F)< \eta .$

We now recall the quantitative version of Alexandrov’s theorem proved in [9, Theorem 1.3], which can be also seen as a Łojasiewicz–Simon inequality with sharp exponents. It will be the main tool to prove the exponential stability of the geometric flows considered. We slightly rephrase the conclusion as it will be more useful in the following.

### Theorem 1.7

[9, Theorem 1.3] Let $E \subset {\mathbb {T}}^N$ be a strictly stable critical set. There exist $\delta ^* \in (0,1/2)$ and $C=C(E)>0$ with the following property :  for any $f\in C^1(\partial E)\cap H^2(\partial E)$ such that $\Vert f\Vert _{C^1(\partial E)}\le \delta ^*$ and satisfying

1.7 $$\begin{aligned} |E_f|=|E|,\quad \left| \int _{\partial E} f\nu _E\,\text {d}{\mathcal {H}}^{N-1}\right| \le \delta ^* \Vert f\Vert _{L^2(\partial E)}, \end{aligned}$$

setting ${\mathscr {H}}_{E_f}(x)=\textrm{H}_{E_f}(x+f(x)\nu _E(x))$ for $x \in \partial E,$ we have

1.8 $$\begin{aligned} \Vert f\Vert _{H^1(\partial E)}\le C\Vert {\mathscr {H}}_{E_f} - \bar{{\mathscr {H}}_{E_f}} \Vert _{L^2(\partial E)}. \end{aligned}$$

### Remark 1.8

Note that Eq. (1.8) in particular implies that, under the hypotheses of Theorem 1.7, for any $\lambda \in {\mathbb {R}}$ it holds

1.9 $$\begin{aligned} \Vert f\Vert _{H^1(\partial E)}\le C\Vert {\mathscr {H}}_{E_f}-\lambda \Vert _{L^2(\partial E)}. \end{aligned}$$

We conclude this section by recalling the Poincaré and Gagliardo–Nirenberg inequalities on smooth hypersurfaces (see [6] for instance).

### Lemma 1.9

Let $\Sigma \subset {\mathbb {T}}^N$ be a smooth closed hypersurface and $f\in H^1(\Sigma ).$ There exists $C=C(\Sigma )>0$ such that

$$\begin{aligned} \Vert f- {\bar{f}}\Vert _{L^2(\Sigma )}\le C \Vert \nabla _\tau f \Vert _{H^1(\Sigma )}, \end{aligned}$$

where we recall $\nabla _\tau f := \nabla f - (\nabla f \cdot \nu _\Sigma ) \nu _\Sigma .$

### Theorem 1.10

Let $\Sigma \subset {\mathbb {T}}^N$ be a smooth closed hypersurface. Let $l, \ m, \ k \in {\mathbb {N}}$ be such that $1\le l <m,$ and let $1\le r\le \infty .$ There exists a constant *C*,  depending on these constants and on $\Sigma ,$ with the following property :  for every $u \in W^{l,p}( \Sigma )$ we have

$$\begin{aligned} \Vert \nabla ^l u(\cdot ,t)\Vert _{L^p(\Sigma )} \le C \Vert u(\cdot ,t) \Vert ^{\theta }_{W^{m,r}(\Sigma )} \Vert u(\cdot ,t) \Vert ^{1-\theta }_{L^{q}(\Sigma )}, \end{aligned}$$

where

$$\begin{aligned} \frac{1}{p}=\frac{l}{N-1}+\theta \left( \frac{1}{r}-\frac{m}{N-1} \right) +(1-\theta ) \frac{1}{q} \end{aligned}$$

for all $\theta \in [l/m,1)$ for which *p* is nonnegative.

### Short-time existence for the mean curvature flow

Given $T>0$ and $E_0 \subset {\mathbb {T}}^N$ an open smooth set, the *volume-preserving mean curvature flow* in [0, *T*) starting from $E_0$ is the family of sets $(E_t)_{0\le t < T}$ whose outer normal velocity is given by

1.10 $$\begin{aligned} V_t(x)= -\textrm{H}_{E_t}(x) +{\bar{\textrm{H}}}_{E_t}, \quad x \in \partial E_t, \ t \in (0,T). \end{aligned}$$

We remark that this equation should be intended as follows: there exist a smooth open set $E \subset {\mathbb {T}}^N$ and a 1-parameter family of smooth diffeomorphism $\Phi _t:\partial E \rightarrow {\mathbb {T}}^N$ given by $\Phi _t(x)=x+u(x,t) \nu _E(x),$ such that $\Phi _0(\partial E)= \partial E_0,$ $\Phi _t(\partial E)=\partial E_t,$ and

$$\begin{aligned} \partial _t u(x,t) \nu (x) \cdot \nu _{E_t}(\Phi _t(x))= -\textrm{H}_{E_t}(\Phi _t(x)) + {\bar{\textrm{H}}}_{E_t}, \quad x \in \partial E, \ t \in (0,T). \end{aligned}$$

Assuming that the flow starting from $E_0$ exists, following classical computations (see for instance [34]) one can deduce that the evolution equation satisfied by *u* is

$$\begin{aligned} \partial _t u = \Delta _{ E} u + \langle A(x,u,\nabla u), \nabla ^2 u \rangle +J(x,u,\nabla u)+\textrm{H}_E, \end{aligned}$$

where $\Delta _{E}$ is the Laplace–Beltrami operator on ${\partial E},$ *A* is a smooth tensor such that $A(\cdot ,0,0)=0,$ and *J* is a smooth function.

In order to prove the stability of such flow, we need the following short-time existence result.

#### Theorem 1.11

Let $\varepsilon >0,$ let $\beta \in (0,1)$ and let $E \subset {\mathbb {T}}^N$ be a smooth open set. There exists $\delta =\delta (\varepsilon ,E,\beta )>0$ with the following property :  if $E_0$ is the normal deformation of *E* induced by $u_0 \in C^{1,1}(\partial E),$ $\Vert u_0\Vert _{C^{1,1}(\partial E)}\le \delta ,$ and $|E_0|=|E|,$ then there exists $T>0,$ which only depends on *E*,  $\beta$ and the bound on $\Vert u_0\Vert _{C^{1,1}(\partial E)},$ such that the volume preserving mean curvature flow $E_t$ starting from $E_0$ exists in [0, *T*),  the sets $E_t$ are normal deformation of *E* induced by $u(\cdot ,t) \in C^{\infty }(\partial E)$ for all $t \in (0,T),$ and

1.11 $$\begin{aligned} \sup _{t\in (0,T)} \Vert u(\cdot ,t)\Vert _{C^{1,\beta }(\partial E)} \le \varepsilon . \end{aligned}$$

Moreover,  for every $k \in {\mathbb {N}},$ there exist two constants $c_k=c_k(N)>0$ and $C_k=C_k(E)>0$ such that

1.12 $$\begin{aligned} \sup _{t\in (0,T)} t^{c_k}\Vert \nabla ^{k+2} u(\cdot ,t) \Vert _{C^0(\partial E)}\le C_{k} (\Vert u_0 \Vert _{C^{1,1}(\partial E)}+1). \end{aligned}$$

We remark that the proof of this result is classical and can be derived from the Schauder estimates for quasi-linear parabolic equations, as *u* solves a lower-order, nonlinear perturbation of the heat equation. In the following section we will provide a brief outline of the proof for an analogous short-time existence result for the surface diffusion flow (see Theorem 1.21). Similar and simplified arguments would prove the previous result for the mean curvature flow, which is a second order flow.

For the sake of completeness, we provide here an alternative proof of Theorem 1.11 which follows from some results found in the literature. Even if these results are shown in the ambient space ${\mathbb {R}}^N,$ the same arguments can be repeated in the flat torus. The first part of the Theorem is the short-time existence result of [15].

#### Theorem 1.12

[15, Main Theorem] Let $E\subset {\mathbb {T}}^N$ be a smooth open set and $\beta \in (0,1).$ There exists $\delta =\delta (E,\beta )>0$ with the following property :  if $E_0$ is the normal deformation of *E* induced by $u_0 \in C^{1,1}(\partial E),$ $\Vert u_0\Vert _{C^{1,1}(\partial E)}\le \delta ,$ and $|E_0|=|E|,$ then there exists $T>0,$ only depending on *E*,  $\beta$ and the bound on $\Vert u_0\Vert _{C^{1,1}(\partial E)},$ such that the volume-preserving mean curvature flow $E_t$ starting from $E_0$ exists in [0, *T*),  and the sets $E_t$ are normal deformations induced by $u(\cdot ,t) \in C^{\infty }(\partial E)$ for all $t \in (0,T).$ Furthermore,  the mapping $(t,E_0)\mapsto E_t$ is a local smooth semiflow on $C^{1,\beta }(E).$

We remark that the local smooth semiflow property in particular implies that $\Vert u(\cdot )\Vert _{C^{1,\beta }}$ depends continuously on $\Vert u_0\Vert _{C^{1,\beta }}$ (see for instance [5, pag. 66]). In particular, for every $\varepsilon >0$ there exists $\delta (E,\varepsilon ,\beta )>0$ and $T(E,\varepsilon ,\beta )>0$ such that if $\Vert u_0\Vert _{C^{1,\beta }}\le \delta$ then

1.13 $$\begin{aligned} \Vert u(\cdot ,t)\Vert _{C^{1,\beta }}\le \varepsilon \quad \text {for every } t\in (0,T). \end{aligned}$$

In order to obtain the higher-order regularity inequalities, we apply some curvature estimates obtained recently in [27].

#### Theorem 1.13

[27, Theorem 1.1] Assume that $E_0\subset {\mathbb {R}}^N$ is an open bounded set satisfying a uniform inner and outer ball condition with radius *r*. Then,  there exists a time $T=T(r,N)>0$ such that the volume preserving mean curvature flow $E_t$ starting from $E_0$ exists in [0, *T*) and it satisfies a uniform inner and outer ball condition of radius *r*/2. Moreover,  it is smooth in (0, *T*) and satisfies for every $k\in {\mathbb {N}}$

1.14 $$\begin{aligned} \sup _{t\in (0,T)} \left( t^k\Vert \textrm{H}_{E_t}\Vert _{H^k(\partial E_t)}^2 \right) \le C_k, \end{aligned}$$

where $C_k$ depends on $k,|E_0|,r.$

Before proving the short time existence result, we remark a classical result concerning the uniform ball condition.

#### Remark 1.14

Let *E* be a smooth set satisfying a uniform ball condition of radius $r_E.$ Then every small $C^{1,1}$-normal deformations of *E* satisfy a uniform ball condition of radius $r\approx r_E.$ Indeed, it is easy to see that if $E_f$ is the normal deformation of *E* induced by $f\in C^{1,1}(\partial E),$ then the Hausdorff distance between *E* and $E_f$ is bounded by $\Vert f\Vert _{C^0(\partial E)}.$ Furthermore, since $\nabla \text {sd}_{E_f}=\nu _{E_f}$ and $\nu _{E_f}$ can be written as

1.15 $$\begin{aligned} \nu _{E_f}=\left( \nu _E-\sum _{i=1}^{N-1} \frac{\nabla f\cdot v_i}{1+\kappa _i f}v_i\right) \left( 1+ \sum _{i=1}^{N-1} \dfrac{(\nabla f\cdot v_i)^2}{(1+\kappa _i f)^2} \right) ^{-1/2}, \end{aligned}$$

where the family $\{v_i\}_{i=1,\ldots ,N-1}$ denotes an orthonormal frame of the tangent space on $\partial E$ (see [9, eq. (3.3)]), by differentiating (1.15) one can see that

$$\begin{aligned} \Vert \text {sd}_{E_f}-\text {sd}_E \Vert _{C^{1,1}(\partial E)}\le C_E \Vert f\Vert _{C^{1,1}(\partial E)}, \end{aligned}$$

which then implies that $E_f\rightarrow E$ in $C^{1,1}$ if $\Vert f\Vert _{C^{1,1}}\rightarrow 0.$ Therefore, by [8, Theorem 2.6] and [8, Remark 2.7] one infers that the radius *r* of the uniform ball condition of the set $E_f$ depends continuously on $\Vert f\Vert _{C^{1,1}}$ when it is small enough. In particular, for every $\varepsilon >0$ there exists $\delta (r_E,\varepsilon )>0$ such that, if $\Vert f\Vert _{C^{1,1}}\le \delta$ then

1.16 $$\begin{aligned} |r_E-r|\le \varepsilon . \end{aligned}$$

#### Proof of Theorem 1.11

By Theorem 1.12 there exist a time $T'>0$ and a family of evolving functions $u(\cdot ,t),$ which are smooth in $(0,T')$ and satisfy the inequality (1.11). The second bound follows from classic elliptic regularity arguments that we now sketch.

Fix $t\in (0,T'),$ from the bound on $\sup _{t \in (0,T')}\Vert u \Vert _{C^{1,\beta }(\partial E)}$ and (up to rotations) for any given point $x=(x',x_{N})\in \partial E$ we can parametrize in a cylinder $C=B'_r(x)\times (-L,L)$ both $\partial E$ and $\partial E_t$ as graphs of smooth functions $g,g_t.$ From Theorem 1.13 there exists a time $T''$ (depending on $E,\delta$ by Remark 1.14) such that the evolving sets $E_t$ satisfy a uniform inner and outer ball condition of radius *r*/2 for any $t \in (0, T'').$ Let us set $T = \min \{T', T''\}.$ From estimate (1.14) we get that

$$\begin{aligned} \textrm{H}_{E_t}=\text {div}\left( \dfrac{\nabla g_t}{\sqrt{1+|\nabla g_t|^2}}\right) = \dfrac{1}{\sqrt{1+|\nabla g_t|}}\left( I-\dfrac{\nabla g_t\otimes \nabla g_t}{1+|\nabla g_t|^2} \right) :\nabla ^2 g_t \end{aligned}$$

is bounded in $L^{2}(B'_r(x'))$ by a constant which depends on $\vert E_0 \vert ,T,r.$ Then, by uniform geometric Calderon–Zygmund inequality (see [12, Section 3] or [3, Lemma 7.2]) we deduce that, for some $\rho <r,$ in the ball $B'_{\rho }(x')$ the function $g_t$ is bounded in $H^{2}(B'_{\rho }(x'))$ by a constant, depending only on the $L^2$-bound on $\textrm{H}_{E_t},$ the norm of the coefficients of the elliptic operator, which are in turn bounded by $\Vert u_0\Vert _{C^{1,1}}$ thanks to the previous step. Iterating this procedure, we bound the higher norms $H^k(B'_{\rho }(x'))$ of $g_t,$ for every $k\in {\mathbb {N}}.$ Then, we conclude by means of Sobolev embeddings and by a covering argument. $\quad \square$

### Short-time existence for the surface diffusion flow

We now consider the evolution called *surface diffusion flow*, defined by

1.17 $$\begin{aligned} V_t(x)=\Delta _{E_t} \textrm{H}_{E_t}(x), \quad x \in \partial E_t, \ t \in (0,T). \end{aligned}$$

As for the mean curvature flow, the equation above means that there exist a smooth open set $E \subset {\mathbb {T}}^N$ and a 1-parameter family of smooth diffeomorphism $\Phi _t:E \rightarrow {\mathbb {T}}^N$ such that $\Phi _t(x)=x+u(x,t) \nu _E(x),$ $\Phi _t(\partial E)=\partial E_t$ and

$$\begin{aligned} \partial _t u(x,t) \nu _E(x) \cdot \nu _{E_t}(\Phi _t(x))= \Delta _{E_t}\textrm{H}_{E_t}(\Phi _t(x)). \end{aligned}$$

Assuming that the diffeomorphisms above exist, arguing as in [34, pag. 21], one can deduce that the evolution equation satisfied by *u* is

1.18 $$\begin{aligned} \partial _t u= & {} - \Delta ^2_{E_t} u - \dfrac{1}{\nu _E\cdot \nu _{E_t}} \Delta _{E_t} (\nu _E\cdot \nu _{E_t} ) \Delta _{E_t} u + \dfrac{1}{\nu _E\cdot \nu _{E_t}}\Delta _{E_t} P(x,u,\nabla u)\nonumber \\= & {} - \Delta ^2_{E_t} u + {{\tilde{J}}}(x,u,\nabla u,\nabla ^2 u,\nabla ^3 u), \end{aligned}$$

where *P* is a smooth function (assuming that *u* and $\nabla u$ are small), the function ${{\tilde{J}}}$ can be written as

$$\begin{aligned} {{\tilde{J}}}(x,u,\nabla u,\nabla ^2 u, \nabla ^3 u)= \langle {{\tilde{B}}}_1, \nabla ^2 u \rangle + \langle {{\tilde{B}}}_2, \nabla ^2 u\otimes \nabla ^2 u \rangle + \langle {{\tilde{B}}}_3, \nabla ^3 u \rangle +{{\tilde{b}}}_4 \end{aligned}$$

and ${{\tilde{B}}}_1,{{\tilde{B}}}_2,{{\tilde{B}}}_3$ and ${{\tilde{b}}}_4$ are tensor-valued, respectively scalar-valued functions depending on $(x,u,\nabla u)$ and smooth if their arguments are small enough. Here, with a little abuse of notation, $\nabla$ denotes the covariant derivative on $\partial E.$

On the other hand, linearizing the Laplace–Beltrami operator yields the evolution equation (compare with [18, Section 3.1])

1.19 $$\begin{aligned} \partial _t u = -\Delta _E^2 u + \langle A(x,u,\nabla u), \nabla ^4 u \rangle + J(x,u,\nabla u,\nabla ^2 u, \nabla ^3 u), \end{aligned}$$

where *A* is a smooth 4th-order tensor, vanishing when both *h* and $\nabla h$ vanish, and *J* is given by

1.20 $$\begin{aligned} J= & {} \langle B_1, \nabla ^3 u \otimes \nabla ^2 u \rangle + \langle B_2, \nabla ^3 u\rangle + \langle B_3, \nabla ^2 u \otimes \nabla ^2 u \otimes \nabla ^2 u \rangle \nonumber \\{} & {} + \langle B_4, \nabla ^2 u \otimes \nabla ^2 u \rangle + \langle B_5, \nabla ^2 u \rangle + b_6, \end{aligned}$$

where $B_i,$ $i=1,\ldots 5$ and $b_6$ are smooth tensor-valued, respectively scalar-valued functions depending on $(x,u,\nabla u).$

In this subsection we want to prove a short-time existence result for the surface diffusion flow, in particular we will obtain a priori estimates that will be used to prove the stability of the flow. We will follow the classical approach of linearization and fixed point to solve the nonlinear evolution problem, and then employ Shauder-type estimates to show higher order regularity of the flow. We will follow closely what has been done in [18], combining it with the results of [24].

To start we recall some classical results concerning the Cauchy problem for the biharmonic heat equation on a smooth Riemannian manifold $\Sigma$ with metric *g*,  which is the solution to the following problem

1.21 $$\begin{aligned} {\left\{ \begin{array}{ll} \partial _t u=-\Delta _\Sigma ^2 u+f(x,t)\quad &{}\text {on }\Sigma \times [0,\infty )\\ u(\cdot ,0)=u_0\quad &{}\text {on }\Sigma , \end{array}\right. } \end{aligned}$$

once the functions $f,u_0$ are assigned.

#### Theorem 1.15

(p. 251, [16, Theorem 2]) Given $( \Sigma , g)$ a smooth Riemannian manifold, there exists a unique biharmonic heat kernel with respect to *g* denoted as $b_g \in C^\infty \big (\Sigma \times \Sigma \times (0, \infty )\big ).$ Moreover let $T>0,$ for any integers $k, p, q \ge 0$ and for any $(x,y, t) \in \Sigma \times \Sigma \times (0,T)$ we have

1.22 $$\begin{aligned} |\partial _t^k \nabla _x^{p} \nabla _y^{q} b_g(x, y, t)|_g \le C t^{- \frac{n + 4k + p+ q}{4}} \exp \{- \delta \big (t^{-\frac{1}{4}} d_g(x, y)\big )^{\frac{4}{3}} \}, \end{aligned}$$

where $|\cdot |_g=\sqrt{g(\cdot ,\cdot )},$ $\nabla _x$ and $\nabla _y$ are covariant derivatives with respect to *g*,  and the constants $C, \delta >0$ depend on *T*,  *g* and $p + q +4k.$

Given the biharmonic heat kernel $b_g \in C^\infty \big (\Sigma \times \Sigma \times (0,\infty )\big )$ on $(\Sigma ,g)$ and a function $u_0 \in C^0(\Sigma ),$ we define for $(x, t) \in \Sigma \times (0,\infty )$

1.23 $$\begin{aligned} S u_0 (x, t) = \int _\Sigma b_g(x, y, t) u_0(y) {\textrm{d}} V_g(y) \end{aligned}$$

where $$ V_g$$ is the Riemannian volume form. Hence, as usual, $$Su_0$$ is the solution to the homogeneous Cauchy problem

1.24 $$\begin{aligned} {\left\{ \begin{array}{ll} \partial _t v+ \Delta _\Sigma ^2 v = 0 &{}\text {on }\Sigma \times (0,+\infty ) \\ v(\cdot ,0)=u_0(\cdot )&{}\text {on }\Sigma . \end{array}\right. } \end{aligned}$$

Moreover, since the biharmonic heat kernel is smooth for every $t>0,$ we get $S u_0 \in C^{\infty }\big (\Sigma \times (0,+\infty )\big ).$ We now collect some results, which are shown in [24], about the solution of (1.21). The following Schauder-type estimates on the solution of the homogeneous problem (1.24) can then be proved, see [24, Theorem 3.8]. In particular, we modify slightly the formulation of the result, to fit our purposes. One can inspect the proof of [24, Theorem 3.8] (see pag. 7487,7489 in particular) to check the result.

#### Theorem 1.16

Suppose $u_0 \in C^{1,1}(\Sigma )$ and fix $T>0.$ Then there exists $C_1(\Sigma ,T)>0$ such that

1.25 $$\begin{aligned} \sup _{t\in (0,T)} \Vert | S u_0 |_g \Vert _{C^{1,1}(\Sigma )}\le C_1 \Vert u_0\Vert _{C^{1,1}(\Sigma )}, \end{aligned}$$

Furthermore,  for any $l, k \in {\mathbb {N}},$ we have

1.26 $$\begin{aligned} \sup _{t \in (0,T)}t^{l + \frac{k}{4}} \bigg \Vert \Big | \left( \partial _t \right) ^l \nabla _g^{k+2} S u_0(t) \Big |_g \bigg \Vert _{C^0(\Sigma )} \le C_{l,k} \Vert u_0\Vert _{C^{1,1}(\Sigma )}, \end{aligned}$$

for some constants $C_{l,k} >0$ depending on *l*,  *k*,  $\Sigma$ and *T*.

In order to study the evolution problem (1.19) we introduce the following two Banach spaces. Fix $0< T < \infty$ and $0< \beta < 1.$ We define

1.27 $$\begin{aligned} Y_T :=\left\{ u \in C^{0}\big (\Sigma \times (0,T) \big ) : \Vert u\Vert _{Y_T} < \infty \right\} , \end{aligned}$$

where

1.28 $$\begin{aligned} \Vert u\Vert _{Y_T}{} & {} :=\sup _{t\in (0,T) } \left( t^{\frac{1}{2}} \Vert u(\cdot ,t)\Vert _{C^0(\Sigma )} + t^{\frac{1}{2} + \frac{\beta }{4}} [u(\cdot ,t)]_{C^{\beta }(\Sigma )} \right) \nonumber \\{} & {} \quad + \sup _{(x,t) \in \Sigma \times (0,T)} \sup _{0< h < T-t} t^{\frac{1}{2} + \frac{\beta }{4}}\frac{|u(x, t+h) - u(x,t)|}{|h|^{\frac{\beta }{4}}} \end{aligned}$$

and $[\cdot ]_{C^\beta }$ is the usual Hölder seminorm. Similarly, we introduce the space

1.29 $$\begin{aligned} X_T :=\left\{ u \in C^0(\Sigma \times (0,T)) : u(\cdot ,t) \in C^4(\Sigma ), \ \Vert u\Vert _{X_T} < \infty \right\} , \end{aligned}$$

where

1.30 $$\begin{aligned} \Vert u\Vert _{X_T}{} & {} :=\sup _{t\in (0,T)} \Big ( \sum _{k=0}^4 t^{- \frac{1}{2} + \frac{k}{4}} \Vert \nabla ^k u(\cdot ,t)\Vert _{C^0(\Sigma )}+ t^{\frac{1}{2}+ \frac{\beta }{4}} [\nabla ^4 u(\cdot ,t)]_{C^{\beta }(\Sigma )}\nonumber \\{} & {} + t^{\frac{1}{2}} \Vert \partial _t u(\cdot ,t)\Vert _{C^{0}(\Sigma )} + t^{\frac{1}{2}+\frac{\beta }{4}} [\partial _t u(\cdot ,t)]_{C^{\beta }(\Sigma )} \Big ) \nonumber \\{} & {} + \sup _{(x,t) \in \Sigma \times (0,T)} \sup _{0< h< T-t} t^{\frac{1}{2}+\frac{\beta }{4}}\frac{|\nabla ^4u (x, t+h)- \nabla ^4 u(x,t)|_g}{|h|^{\frac{\beta }{4}}}\nonumber \\{} & {} + \sup _{(x,t) \in \Sigma \times (0,T)} \sup _{0< h < T-t} t^{\frac{1}{2}+\frac{\beta }{4}}\frac{|\partial _t u (x, t+h)- \partial _t u (x,t)|}{|h|^{\frac{\beta }{4}}}. \end{aligned}$$

#### Proposition 1.17

The spaces $(Y_T, \Vert \cdot \Vert _{Y_T})$ and $(X_T, \Vert \cdot \Vert _{X_T})$ are Banach spaces.

The proof of the completeness of the spaces $Y_T$ and $X_T$ is standard, indeed one can prove directly that all Cauchy sequence converge to a function in the space and the candidate limit is obtained using a diagonal argument.

#### Remark 1.18

Since the norm $\sum _{k=0}^4\Vert \nabla ^k u\Vert _{C^0}$ is equivalent to the norm $\Vert u\Vert _{C^0}+\Vert \nabla ^4u\Vert _{C^0}$ for $C^4(\Sigma ),$ we have that the norm $\Vert \cdot \Vert _{X_T}$ defined in (1.30) is equivalent to the following norm

$$\begin{aligned} \Vert u\Vert _{X_T}^{'} : = \Vert u\Vert _{X_T} + \sum _{k= 0}^3 \sup _{(x,t) \in \Sigma \times (0,T)} \sup _{0< h < T-t} t^{- \frac{1}{2} + \frac{k}{4}+\frac{\beta }{4}}\frac{|\nabla ^k u (x, t+h)- \nabla ^k u(x,t)|_g}{|h|^{\frac{\beta }{4}}}. \end{aligned}$$

Now we study the nonhomogeneous initial value problem

1.31 $$\begin{aligned} {\left\{ \begin{array}{ll} \partial _t u + \Delta _\Sigma ^2 u = f &{}\text {on } \Sigma \times (0,T) \\ u(\cdot , 0) = 0 &{}\text {on } \Sigma , \end{array}\right. } \end{aligned}$$

where *f* is a function on $\Sigma \times (0,T).$ Given the biharmonic heat kernel $b_g \in C^\infty \big (\Sigma \times \Sigma \times (0,T)\big )$ on $(\Sigma ,g),$ the solution (if it exists) to the nonhomogeneous problem (1.31) should be given by Duhamel’s principle

1.32 $$\begin{aligned} V f (x, t) :=\int _{0}^t \int _\Sigma b_g(x, y, t-s) f(y, s) {\textrm{d}} V_g(y) {\textrm{d}} s, \end{aligned}$$

and, for every $\lambda >0,$ $Vf \in C^{\infty }(\Sigma \times (\frac{\lambda }{2},\lambda )).$

We then recall the following fundamental Schauder-type estimates proved in [24] on solutions of (1.31) (see [24, Remark 3.12] for the final comments on the constant *C*).

#### Theorem 1.19

[24, Theorem 3.10] Fix $0< T < \infty ,$ if $f \in Y_T,$ then $Vf \in X_T$ and there exists a constant $C>0$ depending on $\Sigma ,T$ such that

1.33 $$\begin{aligned} \Vert V f\Vert _{X_T} \le C \Vert f\Vert _{Y_T}. \end{aligned}$$

Moreover, equation $(\partial _t + \Delta _\Sigma ^2 ) Vf = f$ holds in the classical sense on $\Sigma \times (0,T)$ and thus $V f\in C^\infty (\Sigma \times (0,T)).$

We now turn our attention to the evolution equation (1.19), and use the results above for the particular choice $\Sigma = \partial E$ with the Riemannian metric induced by the Euclidean one. We consider the map

1.34 $$\begin{aligned} f[u](x):=\langle A(x,u,\nabla u), \nabla ^4 u \rangle + J(x,u,\nabla u,\nabla ^2 u, \nabla ^3 u), \end{aligned}$$

where *A*,  *J* are the operators defined in (1.19). We now provide the fundamentals estimates on *f*[*u*],  which represents the nonlinear error generated linearizing (1.19).

#### Lemma 1.20

For any $\varepsilon ,\,m>0$ there exist $T, \,\delta >0$ depending on $E,\varepsilon$ with the following properties. For every $u_0\in C^{1,1}(\Sigma )$ and $\psi \in X_T$ satisfying $\Vert \psi \Vert _{X_T}\le m$ it holds

1.35 $$\begin{aligned} f[\psi +S u_0]\in Y_T. \end{aligned}$$

Moreover,  if $\Vert u_0\Vert _{C^{1,1}(\Sigma )}\le \delta$ it holds

1.36 $$\begin{aligned} \Vert f[S u_0]\Vert _{Y_T}\le \varepsilon ( \Vert u_0 \Vert _{C^{1,1}(\Sigma )}+ 1). \end{aligned}$$

Finally,  $\psi _1, \psi _2 \in X_{T}$ satisfying $\Vert \psi _i\Vert _{X_{T}}\le m,$ it holds

1.37 $$\begin{aligned} \Vert f[\psi _1+S u_0]-f[\psi _2 + S u_0]\Vert _{Y_{T}} \le \varepsilon \Vert \psi _1-\psi _2 \Vert _{X_{T}}. \end{aligned}$$

#### Proof

Let $T<1$ to be chosen later and fix $\varepsilon ,m>0.$ We prove only equation (1.36), giving a sketch of the proof for (1.37) and (1.35) as they are analogous; we also drop the dependence on the set *E* in the norms. For clarity of exposition, we prove the results for the simplified error term

1.38 $$\begin{aligned} {{\tilde{f}}}[u](x,t):=\langle A(x,u(x,t),\nabla u(x,t)), \nabla ^4 u(x,t) \rangle +\langle B, \nabla ^3 u(x,t)\otimes \nabla ^2 u(x,t) \rangle ,\nonumber \\ \end{aligned}$$

where *B* is a (constant) tensor of the same dimension of $\nabla ^3 u\otimes \nabla ^2 u$ with $\Vert B \Vert < 1.$ The general case is explained in the appendix, but follows by analogous computations. We will also write *A*(*x*, *t*) and assume implicitly the dependence on $u,\nabla u.$

Firstly, we prove (1.36). In what follows we use the short-hand notation $u=S u_0.$ From the definition of ${{\tilde{f}}}[\cdot ]$ we have

1.39 $$\begin{aligned} \Vert {{\tilde{f}}}[u] \Vert _{C^0}\le & {} \Vert A\Vert _{C^0}\Vert \nabla ^4 u\Vert _{C^0}+ \Vert \nabla ^3 u\Vert _{C^0}\Vert \nabla ^2 u\Vert _{C^0},\nonumber \\ {}[{{\tilde{f}}}[u]]_{C^\beta }\le & {} \Vert \nabla ^4 u\Vert _{C^0} \sup _{\tau \in {\mathbb {T}}^N} \left( |\tau |^{-\beta }|A(x+\tau ,t)-A(x,t)|\right) + \Vert A\Vert _{C^0} [\nabla ^4 u]_{C^\beta }\nonumber \\{} & {} + [\nabla ^3 u]_{C^\beta }\Vert \nabla ^2 u\Vert _{C^0} + \Vert \nabla ^3 u\Vert _{C^0}[\nabla ^2 u]_{C^\beta }. \end{aligned}$$

Then, we multiply by $t^{\frac{1}{2}}$ the first equation in (1.39) to get

$$\begin{aligned} t^{\frac{1}{2}}\Vert {{\tilde{f}}}[u] \Vert _{C^0}\le \Vert A\Vert _{C^0} t^{\frac{1}{2}} \Vert \nabla ^4 u\Vert _{C^0}+ t^{\frac{1}{4}} t^{\frac{1}{4}}\Vert \nabla ^3 u\Vert _{C^0} \Vert \nabla ^2 u\Vert _{C^0}. \end{aligned}$$

By (1.26), with the choice of $l=0,$ $k=0,1,2,$ we have that all the terms $t^{\frac{1}{2}} \Vert \nabla ^4 u\Vert _{C^0},$ $t^{\frac{1}{4}}\Vert \nabla ^3 u\Vert _{C^0}$ and $\Vert \nabla ^2 u\Vert _{C^0}$ are bounded by $\Vert u\Vert _{C^{1,1}}$ (times a constant that depends on *E* which we can suppose equal to one for simplicity). We now fix $\delta >0$ sufficiently small, depending on $\varepsilon$ and *E*,  so that $\Vert A\Vert _{C^0}$ is bounded by $\varepsilon ,$ which can be done since *A* is a smooth tensor and $A(\cdot ,0,0)=0.$ Finally, taking *T* small enough, depending on $\varepsilon$ and *E*,  we conclude

$$\begin{aligned} \sup _{t\in (0,T)} t^{\frac{1}{2}}\Vert {{\tilde{f}}}[u] \Vert _{C^0}\le \varepsilon \Vert u_0\Vert _{C^{1,1}}. \end{aligned}$$

Therefore, taking into account the full expression for the error term *f*[*u*] given by (1.34), one can show that

$$\begin{aligned} \sup _{t\in (0,T)} t^{\frac{1}{2}}\Vert f[u] \Vert _{C^0}\le C\varepsilon \left( \Vert u_0\Vert _{C^{1,1}}+1\right) , \end{aligned}$$

where the last constant comes from the term $b_6.$

Concerning the Hölder seminorm in space, we first remark that

$$\begin{aligned} \sup _{\tau \in {\mathbb {T}}^N} \frac{|A(x+\tau ,t)-A(x,t)|}{|\tau |^{\beta }}\le [A(\cdot , u,\nabla u)]_{C^\beta }+ \Vert \partial _2 A \Vert _{C^0}[u]_{C^\beta }+\Vert \partial _3 A \Vert _{C^0}[\nabla u]_{C^\beta }, \end{aligned}$$

where $\partial _2 A$ and $\partial _3 A$ denote the derivative of *A*(*x*, *y*, *z*) with respect to the second and third components. Therefore, employing again the bounds in (1.25) and (1.26) we can bound

1.40 $$\begin{aligned} t^{\frac{1}{2}}\Vert \nabla ^4 u\Vert _{C^0} \sup _\tau \frac{|A(x+\tau ,t)-A(x,t)|}{|\tau |^{\beta }}\le \varepsilon \Vert u_0\Vert _{C^{1,1}}, \end{aligned}$$

where we took $\delta >0$ sufficiently small, depending on $\varepsilon$ and *E*,  such that

$$\begin{aligned}{}[A(\cdot , u,\nabla u)]_{C^\beta }+ \Vert \partial _2 A \Vert _{C^0}[u]_{C^\beta }+\Vert \partial _3 A \Vert _{C^0}[\nabla u]_{C^\beta } \le \varepsilon , \end{aligned}$$

which is possible since *A* is smooth and $A(\cdot ,0,0)=0.$ Thus, multiplying by $t^{\frac{1}{2}+\frac{\beta }{4}}$ the second equation in (1.39) we obtain

1.41 $$\begin{aligned} t^{\frac{1}{2}+\frac{\beta }{4}}[{{\tilde{f}}}[u]]_{C^\beta }\le & {} t^{\frac{\beta }{4}}\varepsilon \Vert u_0\Vert _{C^{1,1}} + \Vert A\Vert _{C^0}t^{\frac{1}{2}+\frac{\beta }{4}} [\nabla ^4 u]_{C^\beta }\nonumber \\{} & {} +t^{\frac{1}{4}} t^{\frac{1}{4}+\frac{\beta }{4}} \Vert \nabla ^3 u\Vert _{C^{\beta }}\Vert \nabla ^2 u\Vert _{C^0} + t^{\frac{1}{4}} t^{\frac{1}{4}}\Vert \nabla ^3 u\Vert _{C^{0}} t^{\frac{\beta }{4}} \Vert \nabla ^2 u\Vert _{C^\beta }.\nonumber \\ \end{aligned}$$

Then, all the terms in (1.41) with the norms of *u* can be bounded employing (1.25) and (1.26), thus we can make the right-hand side above as small as needed taking $T,\delta$ small enough. Analogous calculations show a similar inequality for the complete error term *f*[*u*].

Finally, we show how to bound the Hölder seminorm in time appearing in $\Vert {{\tilde{f}}}[u]\Vert _{Y_T}.$ We fix $t\in (0,T),h\in (0,T-t).$ To ease notation, we omit to write the evaluation at *x* in the following. We have by the very definition of ${{\tilde{f}}}[u](t)$ that

$$\begin{aligned}{} & {} | {{\tilde{f}}}[u](t+h)- {{\tilde{f}}}[u](t)| \\{} & {} \quad \le | \langle A( u( t+h),\nabla u( t+h)), \nabla ^4 u( t+h) \rangle - \langle A( u( t),\nabla u( t)), \nabla ^4 u( t) \rangle | \\{} & {} \qquad + | \langle B,\left( \nabla ^3 u( t+h)\otimes \nabla ^2 u( t+h) \right) \rangle - \langle B, \left( \nabla ^3 u( t)\otimes \nabla ^2 u( t) \right) \rangle |. \end{aligned}$$

Now by the triangular inequality we obtain

1.42 $$\begin{aligned}{} & {} | \langle A(u( t+h),\nabla u( t+h)), \nabla ^4 u( t+h) \rangle - \langle A( u( t),\nabla u( t)), \nabla ^4 u( t) \rangle | \nonumber \\{} & {} \quad \le \Vert A \Vert _{C^0} | \nabla ^4 u(t+h)- \nabla ^4 u(t) | + \Vert \partial _3 A \Vert _{C^0} | \nabla u(t+h)- \nabla u(t) | \Vert \nabla ^4 u(t)\Vert _{C^0}\nonumber \\{} & {} \qquad +\Vert \partial _2 A \Vert _{C^0} | u(t+h)-u(t) | \Vert \nabla ^4 u \Vert _{C^0}, \end{aligned}$$

and analogously

1.43 $$\begin{aligned}{} & {} | \langle B,\left( \nabla ^3 u( t+h)\otimes \nabla ^2 u( t+h) \right) \rangle - \langle B,\left( \nabla ^3 u(x,t)\otimes \nabla ^2 u(x,t) \right) \rangle | \nonumber \\{} & {} \quad \le |\nabla ^3 u(t+h)-\nabla ^3 u(t)|\Vert \nabla ^2 u\Vert _{C^0} +\Vert \nabla ^3 u \Vert _{C^0} |\nabla ^2 u(t+h)-\nabla ^2 u(t)|. \end{aligned}$$

Therefore from formulas (1.42) and (1.43), we obtain

$$\begin{aligned}&|{{\tilde{f}}}[u](t+h)- {{\tilde{f}}}[u](t)| \\&\quad \le \left( \Vert \partial _2 A \Vert _{C^0} |u(t+h)-u(t)|+\Vert \partial _3 A\Vert _{C^0} | \nabla u(t+h)-\nabla u(t)|\right) \Vert \nabla ^4 u(t)\Vert _{C^0}\\&\qquad + \Vert A\Vert _{C^0} |\nabla ^4 u(t+h)-\nabla ^4 u(t)|+ |\nabla ^3 u(t+h)-\nabla ^3 u(t)|\Vert \nabla ^2 u\Vert _{C^0}\\&\qquad +\Vert \nabla ^3 u \Vert _{C^0} |\nabla ^2 u(t+h)-\nabla ^2 u(t)|. \end{aligned}$$

Applying again (1.25), (1.26), and using the smallness of $\Vert A\Vert _{C^0},$ we conclude (1.36) by taking $T,\ \delta$ small enough.

Following the computations above one can easily prove that if $u_0\in C^{1,1}(\Sigma )$ and $\Vert \psi \Vert _{X_T}\le m,$ it holdsThe only difference is that, in addition to (1.25), (1.26) one can directly exploit the definition of $\Vert \cdot \Vert _{X_T}$ to obtain the required bounds. Also the proof for (1.37) is essentially the same, only much more tedious to write. We show the computations only for the term $\sup _{t\in (0,T)} t^{1/2}\Vert \cdot \Vert _{C^0}$ appearing in the norm of $Y_T$ and for the simplified error term (1.38). For $u_i:=\psi _i+Su_0$ we can writeMultiplying the inequality above by $t^{\frac{1}{2}}$ we have

$$\begin{aligned}&t^{\frac{1}{2}}|{{\tilde{f}}}[u_1] - {\tilde{f}}[u_2] |\\&\quad \le \Big ( \Vert \nabla ^4 u_1\Vert _{C^0} \left( t \Vert \partial _1 A\Vert _{C^0} + t^{\frac{3}{4}} \Vert \partial _2 A\Vert _{C^0} \right) + t^{\frac{1}{2} } \left( \Vert A\Vert _{C^0}+ \Vert \nabla ^3 u_1 \Vert _{C^0}\right) \\&\qquad + t^{\frac{1}{4} }\Vert \nabla ^2 u_2 \Vert _{C^0} \Big )\Vert \psi _1-\psi _2\Vert _{X_T}\\&\quad \le t^{\frac{1}{4}}\Big ( t^{\frac{1}{2}}\Vert \nabla ^4 u_1\Vert _{C^0}\Vert A\Vert _{C^1} +\Vert A\Vert _{C^0}+ t^{\frac{1}{4}}\Vert \nabla ^3 u_1 \Vert _{C^0}+|\nabla ^2 u_2 \Vert _{C^0} \Big )\Vert \psi _1-\psi _2\Vert _{X_T}. \end{aligned}$$

Again, by definition of $\Vert \cdot \Vert _{X_T}$ and by (1.25), (1.26) we conclude taking $T,\ \delta$ small enough. $\quad \square$

We are now able to prove a short-time existence result for the surface diffusion evolution. Thanks to the previous lemmas, we provide also higher order regularity estimates depending on the $C^{1,1}$-bound on the initial datum only. The proof follows closely the corresponding one in [18, 24].

#### Theorem 1.21

Let $\varepsilon >0$ and let $E \subset {\mathbb {T}}^N$ be a smooth open set. There exist $\delta =\delta (\varepsilon ,E),$ $T=T(\varepsilon ,E)>0$ with the following property :  if $E_0$ is the normal deformation of *E* induced by $u_0 \in C^{1,1}(\partial E),$ $\Vert u_0\Vert _{C^{1,1}(\partial E)}\le \delta ,$ and $|E_0|=|E|,$ then the surface diffusion flow $E_t$ starting from $E_0$ exists in [0, *T*),  the sets $E_t$ are normal deformations of *E* induced by $u(\cdot ,t) \in C^{\infty }(\partial E)$ for all $t \in (0,T),$ and

1.44 $$\begin{aligned} \sup _{t\in (0,T)} \Vert u\Vert _{C^{2}(\partial E)} \le \varepsilon . \end{aligned}$$

Moreover,  for every $k \in {\mathbb {N}}\setminus \{0\},$ there exist constants $C_k=C_k(\varepsilon , E)>0$ such that

1.45 $$\begin{aligned} \sup _{t\in [\frac{T}{2},T)} \Vert \nabla ^{k+2} u \Vert _{C^0(\partial E)}\le C_{k} (\Vert u_0\Vert _{C^{1,1}(\partial E)}+1). \end{aligned}$$

#### Proof

In this proof we denote by $C>0$ a constant that depends on *N* and *E* and may change from line to line. Fix $\varepsilon >0.$

**Step 1:** We show existence for (1.19) via a fixed point argument. Let $T<1,$ $\delta < 1$ to be chosen later, and let $u_1 \in C^{\infty }((0,T);C^\infty (\partial E))$ be the solution of

$$\begin{aligned} {\left\{ \begin{array}{ll} \partial _t u_1 = -\Delta ^2_E u_1 &{}\text {on }\partial E\times [0,T), \\ u_1(\cdot , 0)=u_0 &{}\text {on }\partial E, \end{array}\right. } \end{aligned}$$

where $u_0 \in C^{1,1}(\partial E)$ is such that $\Vert u_0\Vert _{C^{1,1}(\partial E)} \le \delta .$ The solution exists and it is given by (1.23), that is $u_1=0+Su_0 =:\psi _1 + Su_0.$ Moreover (1.44) and (1.45) are satisfied by $u_1$ thanks to Theorem 1.16, for $\delta$ small enough depending on $\varepsilon .$ Let now $u_2$ be the solution of

$$\begin{aligned} {\left\{ \begin{array}{ll} \partial _t u_2= -\Delta ^2_E u_2 +f[u_{1}] &{}\text {on }\partial E\times [0,T) , \\ u_2(\cdot , 0)=u_0 &{}\text {on }\partial E, \end{array}\right. } \end{aligned}$$

where *f*[*u*] is defined as in (1.34). By (1.23) and (1.32), the unique solution is given by $u_2=Vf[u_1]+Su_0=Vf[Su_0]+Su_0 =:\psi _2 +Su_0.$ Moreover, by Theorem 1.19 and (1.36) we have the estimate

$$\begin{aligned} \Vert \psi _2\Vert _{X_T}\le C\Vert f[Su_0]\Vert _{Y_T}\le C\varepsilon (\Vert u_0\Vert _{C^{1,1}(\partial E)}+1) \le m, \end{aligned}$$

for *m* sufficiently large. We are then led to define an iterative scheme. We set $u_1,u_2$ as above and for $n \ge 3$ we let $u_n$ be the solution to

1.46 $$\begin{aligned} {\left\{ \begin{array}{ll} \partial _t u_n = -\Delta ^2_E u_n +f[u_{n-1}] &{}\text {on }\partial E\times [0,T), \\ u_n(\cdot , 0)=u_0 &{}\text {on }\partial E, \end{array}\right. } \end{aligned}$$

and we split it as $u_n=S u_0+ V f[u_{n-1}]=:\psi _n +Su_0 .$ We will show that the sequence $\psi _n$ is converging in $X_T.$ To do so, assume that $\psi _j \in X_T$ for $j=1, \ldots , n-1$ with

$$\begin{aligned} \Vert \psi _j\Vert _{X_T}\le m. \end{aligned}$$

Then, by Theorem 1.19 and Lemma 1.20 we get $\psi _n \in X_T$ and

1.47 $$\begin{aligned} \Vert \psi _n\Vert _{X_T}&=\Vert V f[u_{n-1}]\Vert _{X_T} \le C \Vert f[u_{n-1}]\Vert _{Y_T} = C \Vert f[\psi _{n-1}+Su_0]\Vert _{Y_T}\nonumber \\&\le C \sum _{j=2}^{n-1} \Vert f[\psi _{j}+S u_0]-f[\psi _{j-1}+S u_0]\Vert _{Y_T} +C\Vert f[Su_0]\Vert _{Y_T} \nonumber \\&\le C \Big (\sum _{j=1}^{n-1} \varepsilon ^{j} \Big )(\Vert u_0\Vert _{C^{1,1}(\partial E)} + 1) \nonumber \\&\le C \varepsilon \Big (1+\sum _{j=1}^{+\infty } \varepsilon ^{j} \Big )(\Vert u_0\Vert _{C^{1,1}(\partial E)} + 1) \nonumber \\&\le C \varepsilon (\Vert u_0\Vert _{C^{1,1}(\partial E)} + 1) \le m. \end{aligned}$$

Moreover, Lemma 1.20 implies that, for $\delta (\varepsilon ,E), \ T(\varepsilon ,E)$ small enough, it holds for all $n\ge 3$

$$\begin{aligned} \Vert \psi _{n+1}-\psi _n \Vert _{X_T}\le \varepsilon \Vert \psi _{n}-\psi _{n-1} \Vert _{X_T}, \end{aligned}$$

therefore $\psi _n$ is a Cauchy sequence and admits a limit point $\psi$ satisfying

1.48 $$\begin{aligned} \Vert \psi \Vert _{X_T}\le C\varepsilon (\Vert u_0\Vert _{C^{1,1}(\partial E)}+1). \end{aligned}$$

We thus showed the existence of a fixed point $u=\psi + Su_0$ for the problem (1.46). Finally, by (1.25) and (1.48) it holds

1.49 $$\begin{aligned} \Vert u \Vert _{C^2(\partial E)}= & {} \Vert \psi + S u_0\Vert _{C^2(\partial E)}\le \Vert \psi \Vert _{X_T}+ \Vert S u_0\Vert _{C^{2}(\partial E)}\nonumber \\\le & {} C\varepsilon (\Vert u_0\Vert _{C^{1,1}(\partial E)}+1). \end{aligned}$$

**Step 2:** By (1.49) we get straightforwardly that (1.45) holds for $k=0,1,2.$ In order to prove (1.45) for $k \ge 3,$ we consider $x\in \partial E$ and we work under local coordinate, $B'_{r}\cong U \subset \partial E$ such that the metric $(g^{ij})_{i,j=1,\ldots , N-1}$ of $\partial E$ satisfies $\frac{1}{2} \delta _{ij}\le g_E^{ij}\le 2 \delta _{ij}.$ Note in particular that the operator $-\Delta ^2_{E}$ is uniformly elliptic in *U*. In the following we identify $B'_r$ and $U\subset \partial E.$ We also set $g_t$ as the metric on $\partial E_t$ (see [34, pag. 20] for details). Observe that *u* restricted to $B'_{r}\times [\frac{T}{2},T)$ is of class $C^{\infty }$ by the previous step. Recalling that $u=\psi +S u_0,$ we have that the function $\psi$ satisfies

1.50 $$\begin{aligned} \partial _t \psi =-\Delta _{g_{t}}^2 \psi + (\partial _t +\Delta ^2_{g_{t}})(S u_0)+f'=: -\Delta _{g_{t}}^2 \psi + {{\tilde{f}}}. \end{aligned}$$

Taking $\nabla _{g}$ in (1.50) shows that the function $\nabla _{g}\psi$ satisfies the equation

1.51 $$\begin{aligned} \partial _t \nabla _{g} \psi= & {} -\Delta _{g_{t}}^2 \nabla _{g} \psi -(\nabla _{g} g^{ij}_{t})g^{kl}_{t} (\psi )_{ijkl}-g^{ij}_{t} (\nabla _{g} g^{kl}_{t})(\psi )_{ijkl} +\nabla _{g}{{\tilde{f}}}\nonumber \\=: & {} -\Delta _{g_{t}}^2 \nabla _{g} \psi + F, \end{aligned}$$

where the error term *F* contains the derivative of $\psi$ up to order four. To estimate $\Vert F\Vert _{C^{\beta /4}([\frac{T}{2},T]; C^\beta (B'_r))}$ we first observe that, by (1.26), it follows

$$\begin{aligned} \Vert \nabla _{g}\left( (\partial _t+\Delta ^2_{g_{t}})(S {u_0})\right) \Vert _{C^{\beta /4}([\frac{T}{2},T); C^\beta (B'_1))}\le C \varepsilon ( \Vert u_0\Vert _{C^{1,1}(\partial E)}+1). \end{aligned}$$

Secondly, we remark that the other terms of *F* can be bounded analogously, recalling that they contain derivatives of $\psi$ up to order four and using (1.48), to show that

1.52 $$\begin{aligned} \Vert F\Vert _{C^{\beta /4}([\frac{T}{2},T); C^\beta (B'_r))} \le C \varepsilon ( \Vert u_0\Vert _{C^{1,1}(\partial E)}+1). \end{aligned}$$

Note now that $\partial _t+\Delta ^2_{g_{t}}$ is a uniformly parabolic operator, since the coefficients of $\Delta ^2_{g_t}$ are close to the ones of $\Delta ^2_E$ depending on $\Vert u(\cdot ,t) \Vert _{C^{1,1}(\partial E)}$ as $g_{E_u}^{ij}-g_{E}^{ij} = B(x,u,\nabla u)$ and *B* is a smooth function with $B(x,0,0)=0,$ see again [34, pag. 20]. Since $\nabla _{g}\psi$ solves (1.51), by the standard interior Schauder estimates and the bound (1.52), there exists $C>0,$ which depends on *T* and thus on $\varepsilon$ and *E*,  such that

$$\begin{aligned} \Vert \nabla _{g}\psi \Vert _{C^{1,\beta /4}([\frac{T}{2}, T); C^{4,\beta }(B'_{ r/2}))}&\le C\left( \Vert F\Vert _{C^{\beta /4}([\frac{T}{4},T); C^\beta (B'_r))} +\Vert \nabla _{g} \psi \Vert _{C^0(B'_r\times [\frac{T}{4},T))}\right) \\&\le C \varepsilon (\Vert u_0\Vert _{C^{1,1}(\partial E)} + 1), \end{aligned}$$

where we noted that $\Vert \psi \Vert _{C^1((B'_r\times [\frac{T}{4},T)))}\le \Vert \psi \Vert _{X_T}$ and employed again (1.48). Finally, we conclude

$$\begin{aligned} \sup _{t \in [\frac{T}{2},T)} \Vert \nabla ^5 u\Vert _{C^0(\partial E)} \le C (\Vert u_0\Vert _{C^{1,1}(\partial E)} +1) . \end{aligned}$$

By induction, one can prove (1.45) for every $k \in {\mathbb {N}}.$ $\quad \square$

## Stability

### Stability of the volume preserving mean curvature flow

In this subsection, we study the evolution by mean curvature (1.10) of normal deformations of a strictly stable set, as defined in Definition 1.1. Suppose that *E* is a strictly stable set and that $E_0=E_{u_0}$ is a smooth normal deformation of *E*. By Theorem 1.11, the volume preserving mean curvature flow starting from $E_0$ exists in a short time interval, and the evolving sets $E_t$ can be parametrized as normal deformations of the set *E* induced by functions $u(\cdot ,t)$ satisfying

$$\begin{aligned} {\left\{ \begin{array}{ll} u_t(x,t) \nu _{E_t}(p)\cdot \nu _E(x)=-\left( \textrm{H}_{E_t}(p)- {{\bar{\textrm{H}}}}_{E_t}\right) \quad x\in \partial E,\\ u(\cdot ,0)=u_0 \end{array}\right. } \end{aligned}$$

where $p= x+u(x,t)\nu _E(x)$ and $.$ The scalar product above (see for instance [9, eq. (3.4)]) can be written as

$$\begin{aligned} \nu _{E_t}(p)\cdot \nu _E(x)=\left( 1+\sum _{j=1}^{N-1}\dfrac{(\partial _{\tau _j} u(x,t))^2}{(1+\kappa _j(x) u(x,t))^2} \right) ^{ -1/2}, \end{aligned}$$

where $\kappa _j(x)$ and $\tau _j(x)$ are, respectively, the principal curvatures and the principal directions of *E* at *x*. In particular, we remark that $\nu _{E_t}(p)\cdot \nu _E(x)=1+O(\Vert u(\cdot ,t)\Vert _{H^1}).$ We can then prove the first part of the main result, that is Theorem 0.1, concerning the long time behaviour of the volume preserving mean curvature flow.

#### Proof of (i) Theorem 0.1

Let $\varepsilon ,\ \delta (\varepsilon ) \in (0,1)$ to be chosen later. In the following, if not otherwise stated, the constants depends on *N*, *E* and may change from line to line. Fix for instance $\beta = 1/2$ and suppose that $\delta$ is smaller than the constant given by Theorem 1.11. We also use the short-hand notation $\pi _f:=(\pi _E|_{E_f})^{-1}.$

**Step 1.** We start by proving that $P(E_t)-P(E)\le C e^{-ct}$ as long as the flow exists.

Let $u_0\in C^{1,1}(\partial E)$ with $\Vert u_0\Vert _{C^{1,1}}\le \delta < 1.$ By Theorem 1.11 there exist a time $T>0 ,$ which depends on *E* and the bound on $\Vert u_0\Vert _{C^{1,1}}< 1,$ and a smooth flow $E_t$ starting from $E_0$ for $t\in [0,T).$ Moreover, $E_t=E_{u(\cdot ,t)}$ and $u(\cdot ,t)$ satisfies (1.11) and (1.12). Without loss of generality we can assume $T<\infty .$

We notice that, considering $\varepsilon ,\delta$ smaller, the value of *T* does not change.

We recall the following well-known identities, holding along the smooth flow

2.1 $$\begin{aligned} \dfrac{\,\text {d}}{\,\text {d}t}|E_t|=0,\quad \dfrac{\,\text {d}}{\,\text {d}t}P(E_t)= -\Vert \textrm{H}_{E_t}-{{\bar{\textrm{H}}}}_{E_t} \Vert _{L^2(\partial E_t)}^2. \end{aligned}$$

Let $\delta ^*$ be the constant given by Theorem 1.7, $p>N-1$ and $\eta =\eta (\delta ^*, p)$ given by Lemma 1.3. By estimates (1.11), (1.12) and by interpolation we have that $\Vert u(\cdot ,t)\Vert _{W^{2,p}(\partial E)}\le \eta$ for every $t\in [T/2,T),$ up to taking $\varepsilon$ smaller and therefore $\delta$ smaller. Thus for any $t\in [T/2,T)$ we can apply Lemma 1.3 to find $\sigma _t\in {\mathbb {T}}^N$ and a function ${\tilde{u}}(\cdot ,t)$ such that $E_t +\sigma _t = E_{{{\tilde{u}}}(\cdot ,t)}$ and

$$\begin{aligned}{} & {} |\sigma _t| \le C \Vert u(\cdot ,t)\Vert _{W^{2,p}(\partial E)},\ \Vert {{\tilde{u}}}(\cdot ,t)\Vert _{W^{2,p}(\partial E)}\le C \Vert u(\cdot ,t)\Vert _{W^{2,p}(\partial E)},\\{} & {} \left|\int _{\partial E_t} {{\tilde{u}}}(\cdot ,t)\nu _{E_t} \right|\le \delta ^*\Vert {{\tilde{u}}}(\cdot ,t) \Vert _{L^2(\partial E)}. \end{aligned}$$

Furthermore, Lemma 1.5 (taking $\delta$ smaller if needed) implies that $\Vert {{\tilde{u}}}(\cdot ,t) \Vert _{C^1(\partial E)}\le \delta ^*.$ We then apply Theorem 1.7 to the set $E_t+\sigma _t$ to obtain

2.2 $$\begin{aligned} \Vert {{\tilde{u}}}(\cdot ,t) \Vert _{H^1(\partial E)}\le C \Vert {\mathscr {H}}_{E_t+\sigma _t}-\lambda \Vert _{L^2(\partial E)} \end{aligned}$$

for any $\lambda \in {\mathbb {R}},$ where we recall ${\mathscr {H}}_{E_t+\sigma _t}(x)=\textrm{H}_{E_t}(x+{{\tilde{u}}}(x)\nu _E(x)).$ From the previous equation, first by the change of variable $y=x+{{\tilde{u}}}(x,t)\nu _E(x)$ (estimating the Jacobian with the bounds on ${{\tilde{u}}}$ and Lemma 1.5), and then by translation invariance, we arrive at

2.3 $$\begin{aligned} \Vert {{\tilde{u}}}(\cdot ,t) \Vert _{H^1(\partial E)}\le C \Vert \textrm{H}_{E_t+\sigma _t}-\lambda \Vert _{L^2(\partial E_t+\sigma _t)} = C \Vert \textrm{H}_{E_t}-\lambda \Vert _{L^2(\partial E_t)}. \end{aligned}$$

We now claim that

2.4 $$\begin{aligned} P(E_t+\sigma _t)-P(E)=P(E_{{{\tilde{u}}}(\cdot ,t)})-P(E)\le C \Vert {{\tilde{u}}}(\cdot ,t)\Vert ^2_{H^1(\partial E)}, \end{aligned}$$

which is a classical result but we provide a proof for the sake of completeness.

Let us define, for every $x \in \partial E,$ the function

$$\begin{aligned} Q(x):=\bigg ( 1+ \sum _{j=1}^{N-1} \frac{(\partial _{\tau _j}{\tilde{u}}(x,t))^2}{(1+\kappa _j(x) {\tilde{u}}(x,t) )^2} \bigg )^{\frac{1}{2}} \end{aligned}$$

where $\tau _1(x),\ldots , \tau _{N-1}(x)$ and $\kappa _1(x),\ldots , \kappa _{N-1}(x)$ are, respectively, the principal directions and curvatures of $\partial E$ at *x*. Then by [9, Lemma 3.1] we have

$$\begin{aligned} P(E_t+ \sigma _t)= & {} P(E_{{\tilde{u}}(\cdot ,t)}) = \int _{\partial E} Q(x)\prod _{i=1}^{N-1}\left( 1+ \kappa _i(x){\tilde{u}}(t,\,x)\right) \,d {\mathcal {H}}^{N-1}(x) \\= & {} P(E)+ \int _{\partial E}\big (\textrm{H}_{E}{\tilde{u}}(\cdot ,t) + O({\tilde{u}}(\cdot ,t)^2)+ O(\vert D {\tilde{u}}(\cdot ,t)\vert ^2) \big )\, d {\mathcal {H}}^{N-1} \\\le & {} P(E)+ \textrm{H}_{E}\int _{\partial E} {\tilde{u}}(\cdot ,t) \, d {\mathcal {H}}^{N-1}+ C\int _{\partial E} \big ({\tilde{u}}(\cdot ,t)^2+ \vert D {\tilde{u}}(\cdot ,t)\vert ^2 \big )\, d {\mathcal {H}}^{N-1}\\\le & {} P(E)+ C \Vert {\tilde{u}}(\cdot ,t) \Vert ^2_{H^1(\partial E)}, \end{aligned}$$

where we have used that $\textrm{H}_E= \sum _{i=1}^{N-1} \kappa _i$ and the inequality

$$\begin{aligned} \left| \int _{\partial E} {\tilde{u}}(\cdot ,t)\, d {\mathcal {H}}^{N-1}\right| \le C \int _{\partial E} {\tilde{u}}(\cdot ,t)^2 \, d {\mathcal {H}}^{N-1}, \end{aligned}$$

which follows from the fact that $|E_t|=|E_0|$ (see [9, Remark 3.2]). Hence, we prove the claim in (2.4).

We now define the Lyapunov functional ${\mathscr {E}}(t)=P(E_t)-P(E),$ which is non increasing by (2.1). Moreover, by translation invariance, from (2.3), (2.4) and for any $\lambda \in {\mathbb {R}}$ we have

2.5 $$\begin{aligned} P(E_t)-P(E)=P(E_t+\sigma _t)-P(E)\le C \Vert \textrm{H}_{E_t}-\lambda \Vert ^2_{L^2(\partial E_t)}. \end{aligned}$$

Since for any $t\in (0,T)$ Eq. (2.5) for the particular choice of $\lambda ={{\bar{\textrm{H}}}}_{E_t}$ implies

$$\begin{aligned} {\mathscr {E}}'(t) = -\Vert \textrm{H}_{E_t}-{{\bar{\textrm{H}}}}_{E_t}\Vert _{L^2(\partial E_t)}^2 \le -C {\mathscr {E}} (t), \end{aligned}$$

by Gronwall’s inequality we conclude (recalling ${\mathscr {E}}(0)\ge {\mathscr {E}}( T/2)$)

2.6 $$\begin{aligned} {\mathscr {E}}(t)\le {\mathscr {E}}(0) e^{-C(t-T/2)}, \quad \forall t\in [T/2,T). \end{aligned}$$

**Step 2.** We now show that the flow exists for every $t\ge 0$ and it converges exponentially fast to *E* up to translations.

Up to taking $\delta$ smaller, we can use the quantitative isoperimetric inequality in Theorem 1.6 to find the existence of translations $\tau _t$ such that

$$\begin{aligned} C|E \triangle (E_t +\tau _t)|^2 \le P(E_t)-P(E) \le P(E_0)-P(E). \end{aligned}$$

Furthermore, since all the evolving sets $\{ E_t\}_{t\in [T/2,T)}$ satisfy a uniform inner and outer ball condition by Remark 1.14, by classical convergence results (see e.g. [8, Theorem 3.2]) we have that $E_t+\tau _t$ is $C^{1}-$close to *E*. In particular, there exist smooth (by the implicit map theorem) functions $v(\cdot ,t): \partial E \rightarrow {\mathbb {R}}$ such that $E_t+\tau _t=E_{v(\cdot ,t)}$ and

$$\begin{aligned} |\tau _t| \le \max _{x\in \partial E_t+\sigma _t} \text {dist}_{\partial E_t}(x) \le \Vert u(\cdot ,t)\Vert _{C^0(\partial E)} +\Vert v(\cdot ,t)\Vert _{C^{0}(\partial E)}\le 2 \varepsilon , \end{aligned}$$

up to taking $\delta$ smaller. Therefore, recalling (2.6), we have

2.7 $$\begin{aligned} \Vert v(\cdot ,t)\Vert _{L^1(\partial E)}^2\le C(P(E_0)-P(E)) e^{-C(t-T/2)}. \end{aligned}$$

By Lemma 1.5, we also have $\Vert v(\cdot ,t)\Vert _{C^k(\partial E)}\le C( \Vert u(\cdot ,t)\Vert _{C^k(\partial E)}+|\tau _t|)$ for every $k\ge 2.$ For every $t\in [T/2,T),$ by combining the previous estimate with (1.12), (2.7) and interpolation inequalities, for any $l\in {\mathbb {N}}$ there exist $k(l)\in {\mathbb {N}}, \theta (l)\in (0,1)$ and $C=C(E,l)>0$ such that

2.8 $$\begin{aligned} \Vert \nabla ^l v(\cdot ,t)\Vert _{C^{0}}\le C \Vert v(\cdot ,t)\Vert _{L^1}^{\theta } \Vert v(\cdot ,t) \Vert _{C^k}^{1-\theta }\le {C}{T^{-\frac{k}{4}(1-\theta )}}(P(E_0)-P(E))^{\frac{\theta }{2}} e^{-C (t-T/2)}.\nonumber \\ \end{aligned}$$

Choosing ${\mathscr {E}}(0)=P(E_0)-P(E)$ small (hence choosing $\delta$ small) we can then apply again Theorem 1.11 with the new initial set $E_{v(\cdot ,T/2)}=E_{T/2} +\tau _{T/2}$ to get existence of the translated flow up to the time 3*T*/2. We remark that, by uniqueness, the flow above is well defined since it coincides in [*T*/2, *T*) with the flow $E_t$ translated by $\tau _t$ and estimate (2.6) now holds for all $t\in [T/2,3T/2).$ Since now the bound (2.8) is uniform along the flow, choosing at every step the times $t=n T/2,$ we can iterate the procedure above to prove that the flow exists for all times $t\in [0,\infty ).$ Moreover, for every $t\in (0,\infty )$ there exists a translation $\tau _t$ such that $E_t+\tau _t=E_{v(\cdot ,t)}$ with *v* satisfying (2.8). In particular, we have that $v\rightarrow 0$ exponentially in $C^k$ for any *k*,  as $t\rightarrow \infty$ and thus $E_t+\tau _t\rightarrow E$ in $C^k$ for every *k*. This also implies (reasoning as in (2.3)) that $\Vert \textrm{H}_{E_t}-{{\bar{\textrm{H}}}}_{E_t} \Vert _{L^2(\partial E)}\rightarrow 0$ exponentially fast.

**Step 3.** We conclude by showing the convergence of the whole flow to a translate of *E*.

Let us prove the convergence of the translations $\{\tau _t\}_{t\ge 0}.$ By compactness we can find a sequence $t_n\rightarrow \infty$ such that $\tau _{t_n} \rightarrow \tau .$ Defining

2.9 $$\begin{aligned} {\mathcal {D}}(F,G):=\int _{ F\triangle G}\text {dist}_{\partial G}(x)\,\text {d}x, \end{aligned}$$

following the computations of [2, pag. 21] we see

2.10 $$\begin{aligned} \left| \dfrac{\,\text {d}}{\,\text {d}t}{\mathcal {D}}(E_t, E-\tau )\right|= & {} \left| \dfrac{\,\text {d}}{\,\text {d}t} \int _{E_t\triangle (E-\tau )}\text {dist}_{\partial E\tau _t}(x) \,\text {d}x\right| \nonumber \\= & {} \left| \int _{E_t} \text {div}(\text {sd}_{E-\tau }(x) V_t(x) \nu _{E_t}(x)) \,\text {d}x\right| \nonumber \\= & {} \left| -\int _{\partial E_t} \text {sd}_{ E-\tau }(x) (\textrm{H}_{E_t}(x) -{{\bar{\textrm{H}}}}_{E_t}(x)) \,\text {d}{\mathcal {H}}^{N-1}(x)\right| \nonumber \\\le & {} P(E_0)\Vert \textrm{H}_{E_t}-{{\bar{\textrm{H}}}}_{E_t}\Vert _{L^2(\partial E)} \left( \sup _{x \in \partial E_t}\text {dist}_{\partial E-\tau }(x) \right) \nonumber \\\le & {} C e^{-Ct} \left( \sup _{x \in {\mathbb {T}}^N}\text {dist}_{\partial E-\tau }(x) \right) \le C e^{-Ct}, \end{aligned}$$

where we recall that $V_t$ is the velocity of the flow in the normal direction (see (1.10)). Clearly, condition (2.10) implies that ${\mathcal {D}}(E_t, E-\tau )$ admits a limit as $t\rightarrow +\infty .$ By the previous step and since $\tau _{t_n}\rightarrow \tau ,$ we deduce that

$$\begin{aligned} {\mathcal {D}}(E_t, E-\tau )\rightarrow 0 \quad \text {as }t\rightarrow +\infty . \end{aligned}$$

Assume now that $\sigma \in {\mathbb {T}}^N$ is the limit of $\tau _{s_n}$ along a subsequence $s_n\rightarrow \infty$ as $n\rightarrow +\infty .$ By the previous step, $E_{s_n}\rightarrow E-\sigma ,$ therefore

$$\begin{aligned} 0=\lim _{n \rightarrow + \infty } {\mathcal {D}}(E_{s_n}, E-\tau )={\mathcal {D}}(E-\sigma , E-\tau ), \end{aligned}$$

which implies $\sigma =\tau$ by definition (2.9). This concludes the proof as the exponential convergence follows from Step 2. $\quad \square$

### Stability of the surface diffusion flow

We now focus on surface diffusion flow, which we defined in (1.17). As in the previous subsection, we consider *E* a strictly stable set and $E_0=E_{u_0}$ a smooth normal deformation of *E*. By Theorem 1.21, the surface diffusion flow starting from $E_0$ exists smooth in an interval [0, *T*),  moreover the evolving sets $E_t$ can be written as normal deformations of *E* induced by functions $u(\cdot ,t)$ satisfying

$$\begin{aligned} {\left\{ \begin{array}{ll} u_t(x,t) \nu _{E_t}(p)\cdot \nu _E(x)=\Delta _{ E_t}\textrm{H}_{E_t}(p) \quad \forall x\in \partial E,\\ u(x,0)=u_0(x) \end{array}\right. } \end{aligned}$$

where $$p= x+u(x,t)\nu _E(x).$$

Now, we aim to show the stability result (*ii*) of Theorem 0.1 for the surface diffusion flow. Due to the similarity of the arguments needed with those employed to prove item (*i*) of Theorem 0.1, we will only highlight the main differences between the two.

#### Proof of (ii) Theorem 0.1

Firstly, Theorem 1.21 ensures the existence of a smooth flow $E_t$ for $t\in (0,T)$ of normal deformations of *E* induced by functions $u(\cdot ,t)\in C^{\infty }(\partial E)$ and satisfying (1.44) and (1.45). We recall the following identities, holding along the flow $E_t$ as long as it exists smooth,

2.11 $$\begin{aligned} \dfrac{\,\text {d}}{\,\text {d}t}|E_t|=0,\quad \dfrac{\,\text {d}}{\,\text {d}t}P(E_t)=\int _{\partial E}\textrm{H}_{E_t}(x)\Delta _{E_t} \textrm{H}_{E_t}(x)\, dx= -\Vert \nabla \textrm{H}_{E_t}\Vert _{L^2(\partial E_t)}^2\le 0. \nonumber \\ \end{aligned}$$

Denoting by $C_{E_t}$ the constant in the Poincaré inequality of Lemma 1.9, we get

$$\begin{aligned} \Vert \textrm{H}_{E_t}-{{\bar{\textrm{H}}}}_{E_t} \Vert _{L^2(\partial E_t)} \le C_{E_t}\Vert \nabla \textrm{H}_{E_t}\Vert _{L^2(\partial E_t)}. \end{aligned}$$

Combining the previous inequality with (2.11), we obtain

$$\begin{aligned} \dfrac{\,\text {d}}{\,\text {d}t}P(E_t)\le - C_{E_t}\Vert \textrm{H}_{E_t}-{{\bar{\textrm{H}}}}_{E_t} \Vert ^2_{L^2(\partial E_t)}.\nonumber \\ \end{aligned}$$

Since $\Vert u(\cdot ,t)\Vert _{C^{1,1}(\partial E)}\le c$ for every $t\in (0,T),$ the Poincaré constants $C_{E_t}$ are uniformly bounded in the same time interval and the bound depends on $E,\Vert u\Vert _{C^{1,1}(\partial E)}$ (see e.g. the results in [12]). Thus, we obtain the estimate $\frac{\,\text {d}}{\,\text {d}t}P(E_t)\le -C\Vert \textrm{H}_{E_t}-{{\bar{\textrm{H}}}}_{E_t}\Vert ^2_{L^2(\partial E_t)}$ uniformly in (0, *T*). We then conclude by following the same arguments of part (*i*). $\quad \square$

## Data Availability

This paper has no associated data.

## Appendix A: Sketch of a general proof of the Lemma 1.20

In this appendix we complete the proof of Lemma 1.20 in the general case, i.e. considering the full nonlinear error term given by (1.20).

### Proof

As in Lemma 1.20, let $T<1$ to be chosen later. We prove only Eq. (1.36), since the proof of the Eqs. (1.37), (1.35) is completely analogous.

We set

$$\begin{aligned}{} & {} f[u](x,t):=\langle A(x,u(x,t),\nabla u(x,t)), \nabla ^4 u(x,t) \rangle \\{} & {} + J(x,u (x,t),\nabla u (x,t),\nabla ^2 u (x,t), \nabla ^3 u (x,t)). \end{aligned}$$

Since the estimates for the first term of *f*[*u*] have been presented in the proof of Lemma 1.20, we focus on bounding the terms of $J(x,u,\nabla u,\nabla ^2 u, \nabla ^3 u)$ with respect to the norm $\Vert \cdot \Vert _{C^0}.$ Considering the term $\langle B_1, \nabla ^3 u \otimes \nabla ^2 u \rangle ,$ we have

$$\begin{aligned} \langle B_1, \nabla ^3 u \otimes \nabla ^2 u \rangle \le \Vert B_1 \Vert _{C^0} \Vert \nabla ^3 u \otimes \nabla ^2 u \Vert _{C^0} \le C \Vert \nabla ^3 u \Vert _{C^0} \Vert \nabla ^2 u \Vert _{C^0}, \end{aligned}$$

as long as $\Vert u\Vert _{C^1}$ is small. Hence, with the same arguments presented for the functional $\langle B, \nabla ^3 u\otimes \nabla ^2 u\rangle$ we obtain

$$\begin{aligned} \sup _{t \in (0,T)} t^{\frac{1}{2}}\langle B_1, \nabla ^3 u \otimes \nabla ^2 u \rangle \le \varepsilon \Vert u_0 \Vert _{C^{1,1}} \end{aligned}$$

by choosing $T=T(\varepsilon )$ small enough. We analogously treat the other terms, so we have

$$\begin{aligned} \langle B_2, \nabla ^3 u\rangle \le \Vert B_2 \Vert _{C^0} \Vert \nabla ^3 u \Vert _{C^0} ,\, \langle B_3, \nabla ^2 u \otimes \nabla ^2 u \otimes \nabla ^2 u \rangle \le \Vert B_3 \Vert _{C^0} \Vert \nabla ^2 u \Vert _{C^0}^3 \end{aligned}$$

and

$$\begin{aligned} \langle B_4, \nabla ^2 u \otimes \nabla ^2 u \rangle \le \Vert B_4 \Vert _{C^0} \Vert \nabla ^2 u \Vert _{C^0}^2 ,\, \langle B_5, \nabla ^2 u \rangle \le \Vert B_5 \Vert _{C^0} \Vert \nabla ^2 u \Vert _{C^0}. \end{aligned}$$

Following the arguments of Lemma 1.20, we obtain

$$\begin{aligned}{} & {} \sup _{t \in (0,T)} t^{\frac{1}{2}} \langle B_2, \nabla ^3 u\rangle \le \varepsilon \Vert u_0 \Vert _{C^{1,1}},\\{} & {} \sup _{t \in (0,T)} t^{\frac{1}{2}} \langle B_3, \nabla ^2 u \otimes \nabla ^2 u \otimes \nabla ^2 u \rangle \le \varepsilon \Vert u_0 \Vert _{C^{1,1}},\\{} & {} \sup _{t \in (0,T)} t^{\frac{1}{2}} \langle B_4, \nabla ^2 u \otimes \nabla ^2 u \rangle \le \varepsilon \Vert u_0 \Vert _{C^{1,1}} \end{aligned}$$

and

$$\begin{aligned} \sup _{t \in (0,T)} t^{\frac{1}{2}} \langle B_5, \nabla ^2 u \rangle \le \varepsilon \Vert u_0 \Vert _{C^{1,1}}. \end{aligned}$$

In the end we have that $\Vert b_6\Vert _{C^0}\le C=C(E).$

Therefore, taking *T* small we obtain

$$\begin{aligned} \sup _t t^{\frac{1}{2}}\Vert b_6\Vert _{C^0}\le \varepsilon . \end{aligned}$$

We now focus on the Hölder seminorm in space. We present the calculations only for $\langle B_1, \nabla ^3 u \otimes \nabla ^2 u \rangle ,$ being the other analogous. A straightforward computation shows (using the triangular inequality) that

$$\begin{aligned}{} & {} | \langle B_1(x+h, u(x+h), \nabla u(x+h)), \nabla ^3 u (x+h)\otimes \nabla ^2 u(x+h) \rangle \\{} & {} \qquad - \langle B_1 (x, u(x), \nabla u(x)), \nabla ^3 u (x)\otimes \nabla ^2 u(x) \rangle | \\{} & {} \quad \le (|h | \Vert \partial _1 B_1\Vert _{C^0}+ | u(x+h)-u(x)| \Vert \partial _2 B_1 \Vert \\{} & {} \qquad + | \nabla u(x+h)- \nabla u(x) | \Vert \partial _3 B_1 \Vert _{C^0} ) \Vert \nabla ^3 u\Vert _{C^0}\Vert \nabla ^2 u \Vert _{C^0} \\{} & {} \qquad + \Vert B_1 \Vert _{C^0} ( |\nabla ^3(x+h)-\nabla ^3 u(x) | \Vert \nabla ^2 u \Vert _{C^0}+ \Vert \nabla ^2 u\Vert _{C^0} | \nabla ^2 u(x+h)- \nabla ^2 u(x) |). \end{aligned}$$

Therefore, as in the case $J(x,u,\nabla u, \nabla ^2 u, \nabla ^3 u)= \langle B, \nabla ^3 u \times \nabla ^2 u\rangle ,$ using formula (1.25) and (1.26) we obtain the thesis.

Finally, we show how to bound the Hölder seminorm in time appearing in $\Vert f[u]\Vert _{Y_T}.$ We fix $t\in (0,T),\tau \in (0,T-t)$ and, for simplicity, we omit the dependence on *x*. For the first term, we have

$$\begin{aligned}{} & {} | \langle B_1, \nabla ^3 u (t+\tau ) \otimes \nabla ^2 u (t+\tau )\rangle - \langle B_1, \nabla ^3 u (t)\otimes \nabla ^2 u (t) \rangle | \\{} & {} \quad \le \Vert B_1 \Vert _{C^0} \left[ | \nabla ^3 u(t+\tau )- \nabla ^3 u(t) | \Vert \nabla ^2 u \Vert _{C^0}+ | \nabla ^2 u(t+\tau )- \nabla ^2 u(t)| \Vert \nabla ^3 u \Vert _{C^0}\right] \\{} & {} \qquad +\big (\Vert \partial _2 B_1 \Vert _{C^0} | u(t)-u(t+\tau ) |+ \Vert \partial _3 B_1 \Vert | \nabla u(t)- \nabla u(t+\tau ) | \big ) \Vert \nabla ^3 u\Vert _{C^0} \Vert \nabla ^2 u\Vert _{C^0}. \end{aligned}$$

Then, for the second, third and fourth terms we get, respectively,

$$\begin{aligned}{} & {} | \langle B_2,\nabla ^3 u(t+\tau )\rangle - \langle B_2, \nabla ^3 u(t)\rangle | \le \Vert B_2 \Vert _{C^0} | \nabla ^3 u(t+\tau )- \nabla ^3 u(t) | \\{} & {} \qquad +\big (\Vert \partial _2 B_2 \Vert _{C^0} | u(t)-u(t+\tau ) |+ \Vert \partial _3 B_2 \Vert | \nabla u(t)- \nabla u(t+\tau ) | \big ) \Vert \nabla ^3 u\Vert _{C^0},\\{} & {} | \langle B_3, \nabla ^2 u (t+\tau ) \otimes \nabla ^2 u (t+\tau ) \otimes \nabla ^2 u (t+\tau ) \rangle -\langle B_3, \nabla ^2 u (t)\otimes \nabla ^2 u (t) \otimes \nabla ^2 u (t) \rangle | \\{} & {} \quad \le 3 \Vert B_3\Vert _{C^0} | \nabla ^2 u(t+\tau )- \nabla ^2 u(t) | \Vert \nabla ^2 u \Vert _{C^0}^2\\{} & {} \qquad +\big (\Vert \partial _2 B_3 \Vert _{C^0} | u(t)-u(t+\tau ) |+ \Vert \partial _3 B_3 \Vert | \nabla u(t)- \nabla u(t+\tau ) | \big ) \Vert \nabla ^2 u\Vert _{C^0}^3, \end{aligned}$$

and

$$\begin{aligned}{} & {} |\langle B_4, \nabla ^2 u(t+\tau ) \otimes \nabla ^2 u(t+\tau ) \rangle - \langle B_4, \nabla ^2 u(t) \otimes \nabla ^2 u(t) \rangle |\\{} & {} \quad \le 2 \Vert B_4 \Vert _{C^0} \Vert \nabla ^2 u \Vert _{C^0} |\nabla ^2 u(t+\tau )-\nabla ^2 u(t) |\\{} & {} \qquad +\big (\Vert \partial _2 B_4 \Vert _{C^0} | u(t)-u(t+\tau ) |+ \Vert \partial _3 B_4 \Vert | \nabla u(t)- \nabla u(t+\tau ) | \big ) \Vert \nabla ^2 u\Vert _{C^0}^2. \end{aligned}$$

Finally, for the last two terms we have

$$\begin{aligned}{} & {} | \langle B_5, \nabla ^2 u (t+\tau )\rangle - \langle B_5, \nabla ^2 u (t)\rangle | \le \Vert B_5 \Vert _{C^0} | \nabla ^2 u(t+\tau )- \nabla ^2 u(t)|\\{} & {} \qquad +\big (\Vert \partial _2 B_5 \Vert _{C^0} | u(t)-u(t+\tau ) |+ \Vert \partial _3 B_5 \Vert | \nabla u(t)- \nabla u(t+\tau ) | \big ) \Vert \nabla ^2 u\Vert _{C^0} \end{aligned}$$

and

$$\begin{aligned}{} & {} | b_6(\cdot , u(t+\tau ), \nabla u(t+\tau ))- b_6(\cdot , u(t),\nabla u(t)) |\\{} & {} \quad \le (\Vert \partial _2 b_6 \Vert _{C^0}+ \Vert \partial _3 b_6 \Vert _{C^0})( | u(t+\tau )-u(t) |+ | \nabla u(t+\tau )-\nabla u(t) |). \end{aligned}$$

Therefore, we can conclude with the same arguments used for $\langle B, \nabla ^3 u\otimes \nabla ^2 u\rangle .$ $\square$
